# Genome-Wide Identification and Comprehensive Expression Profiling of Ribosomal Protein Small Subunit (RPS) Genes and their Comparative Analysis with the Large Subunit (RPL) Genes in Rice

**DOI:** 10.3389/fpls.2017.01553

**Published:** 2017-09-15

**Authors:** Anusree Saha, Shubhajit Das, Mazahar Moin, Mouboni Dutta, Achala Bakshi, M. S. Madhav, P. B. Kirti

**Affiliations:** ^1^Department of Plant Sciences, University of Hyderabad Hyderabad, India; ^2^Department of Biotechnology, Indian Institute of Rice Research Hyderabad, India

**Keywords:** rice, ribosomal protein small subunit (RPS) genes, ribosomal proteins, stress responses, gene expression

## Abstract

Ribosomal proteins (RPs) are indispensable in ribosome biogenesis and protein synthesis, and play a crucial role in diverse developmental processes. Our previous studies on Ribosomal Protein Large subunit (RPL) genes provided insights into their stress responsive roles in rice. In the present study, we have explored the developmental and stress regulated expression patterns of Ribosomal Protein Small (RPS) subunit genes for their differential expression in a spatiotemporal and stress dependent manner. We have also performed an *in silico* analysis of gene structure, *cis*-elements in upstream regulatory regions, protein properties and phylogeny. Expression studies of the 34 RPS genes in 13 different tissues of rice covering major growth and developmental stages revealed that their expression was substantially elevated, mostly in shoots and leaves indicating their possible involvement in the development of vegetative organs. The majority of the RPS genes have manifested significant expression under all abiotic stress treatments with ABA, PEG, NaCl, and H_2_O_2_. Infection with important rice pathogens, *Xanthomonas oryzae* pv. *oryzae* (*Xoo*) and *Rhizoctonia solani* also induced the up-regulation of several of the RPS genes. *RPS4, 13a, 18a*, and *4a* have shown higher transcript levels under all the abiotic stresses, whereas, *RPS4* is up-regulated in both the biotic stress treatments. The information obtained from the present investigation would be useful in appreciating the possible stress-regulatory attributes of the genes coding for rice ribosomal small subunit proteins apart from their functions as house-keeping proteins. A detailed functional analysis of independent genes is required to study their roles in stress tolerance and generating stress- tolerant crops.

## Introduction

Ribosomal proteins (RPs) constitute the protein part of the ribosomes and have a significant role in ribosome biogenesis, protein synthesis, cell growth, development, and apoptosis (Ramakrishnan and White, 1998; Naora and Naora, 1999; Maguire and Zimmermann, 2001; Wang Z. et al., 2013). RPs have long been known primarily for their housekeeping functions. But, there are several reports on their extra-ribosomal functions in animal systems (Warner and McIntosh, 2009). The knockdown of individual RPs leads to P53 accumulation, cell death and certain developmental abnormalities in Zebra fish (Uechi et al., 2006; Danilova et al., 2008). Mutations in certain RPs were associated with Diamond-Blackfan Anemia (DBA) and increased risk of cancer (Gazda et al., 2008). On the contrary, specific RPs have been used as therapeutic targets in cancer treatment. Overexpression of RPS2 has been linked with the survival of prostate tumors, whereas suppression of RPS2 or RPS3a resulted in apoptosis (Wang et al., 2009).

The number of RPs varies within eukaryotes. In yeast 78 RPs (32 of the small subunit and 46 of large subunit) are encoded by 137 genes (Planta, 1997). Two hundred and forty nine genes encode the 80 RPs of *Arabidopsis*; 32 are small subunit, and 48 are large subunit proteins (Barakat et al., 2001; Wang J. et al., 2013). In rice, 34 large subunit proteins are encoded by 123 genes (Moin et al., 2016b) showing that multiple copies of genes encode an individual ribosomal protein in eukaryotes (McIntosh and Bonham-Smith, 2006). This can be correlated with the requirement of ample supply of RPs during rapid growth phase (Filipowicz and Hohn, 2012). Most of these genes are specifically expressed during a certain developmental stage or in a particular tissue, or they may be functionally redundant. The subsequent lack of sufficient quantity of individual RPs can lead to non-lethal phenotypic abnormalities and aberrant translational efficiency (Hulm et al., 2005; Komili et al., 2007; Degenhardt and Bonham-Smith, 2008).

Several studies have demonstrated important roles of RPs in development and physiology of organisms as their reduced level in the organisms resulted in detrimental effects. Semi-dominant mutation of cytoplasmic ribosomal protein RPL27aC resulted in multiple developmental defects such as changes in leaf patterning, inflorescence, floral meristem function and seed set (Szakonyi and Byrne, 2011). In *Arabidopsis*, PFL codes for the ribosomal protein S18 and a mutation at PFL locus resulted in developmental abnormalities involving reduced fresh weight, pointed first leaves and stunted growth indicating the importance of the corresponding protein in meristem activity (Van Lijsebettens et al., 1994). RPL18aB is presumed to have a critical role in male gametophyte development and embryo pattern formation in *Arabidopsis* with the *rpl18aB* mutants exhibiting defects in pollen growth (Yan et al., 2016). Genes coding for cytoplasmic ribosomal proteins, L10a, L9, and L5 play an important role in determining the adaxial and abaxial fate of leaf during development via their interaction with adaxial *HD-ZIPIII* gene, *REVOLUTA*, and abaxial *KANADI* gene (Pinon et al., 2008). Aberrant expression of ribosomal protein S6 in *Arabidopsis* leads to a deleterious effect on survival of cells indicating their role in a complex translational regulation. Insertion of ribosomal protein S6 gene in antisense orientation resulted in over-expression of antisense ribosomal protein S6 RNA, which led to a reduced level of *rps6* expression. This might be the reason for subdued expression of some other specific proteins, which is manifested in the form of complex phenotypic disorders including multiple leaves and flower formation in close vicinity (Morimoto et al., 2002). In *Saccharomyces cerevisiae*, RPs have been predicted to participate in several extra-ribosomal functions, which are related to ribosome assembly, replicative life span, DNA repair, adhesive growth and filament formation (Lu et al., 2015).

T-DNA insertional mutagenesis in one of the RPS27 genes (*AtRS27A)* led to strong growth inhibition and the formation of tumor-like structure in place of auxiliary roots in the presence of methyl methanesulfonate (MMS) in growth medium (Revenkova et al., 1999). In *Arabidopsis*, delayed cell division and complete developmental arrest was observed in *RPS5* hetero- and homozygous mutants, respectively (Weijers et al., 2001). The mutation in a key translation initiation factor, RPL24 in *Arabidopsis* resulted in perturbed gynoecium development with diminished ovary and lengthened gynophore (Nishimura et al., 2004).

In addition to their crucial roles in developmental processes in plants, several reports also focused on their expression pattern in response to several external stimuli. The expression of several RP genes is also regulated by phytohormones, ABA and cytokinin. ABA negatively regulates the expression of *RPS14, RPS16, RPS13a*, and *RPL30*, whereas cytokinin induced the expression of these genes at both the transcript and protein levels (Cherepneva et al., 2003). In rice, *RPS4, 7, 8, 9, 10, 19, 26*, and *RPL2, 5, 18, 44* were among the early responsive genes that were up-regulated under salt stress (Kawasaki et al., 2001). Nutrition deficiency also resulted in the differential expression pattern of RP genes in *Arabidopsis*. Comparison of differentially expressed RP genes under Fe and Pi deficiency in *Arabidopsis* roots revealed that both the RPS and Ribosomal Protein Large subunit (RPL) genes were differentially expressed under Fe deficiency, whereas only RPL genes were differentially expressed under Pi deficiency (Wang J. et al., 2013). Induction of soybean RPs, RPS13, RPS6, and RPL37 in response to cold treatment gave a clue to their probable role in secondary signaling under low-temperature conditions (Kim et al., 2004). These reports point toward the differential expression of RP genes in response to stress treatments leading to differential accumulation of RPs in the ribosomal apparatus, which might help ribosome remodeling and selective translation to cope up with nutrition deficiency or other unfavorable conditions (Wang J. et al., 2013).

Screening of a large-pool of activation tagged mutant population of rice for high water-use efficiency revealed the significant upregulation of RPL genes (*6* and *23a*) in our previous studies (Moin et al., 2016a). This subsequently persuaded us to study the regulatory role of all the RPL genes under different external stimuli (Moin et al., 2016b). This study led another group to identify the insect resistance roles of *NlRPL5* in rice as an important responsive gene in resistance to the brown plant hopper, *Nilaparvata lugens* (Zhu et al., 2017). The objective of the present study is to ascertain whether RPS genes are stress responsive like the RPL genes (Moin et al., 2016b).

Hence, we have attempted to analyse the differential expression patterns of ribosomal protein small subunit genes (RPS genes, one from each family) for their spatiotemporal and stress induced regulation along with a comparative analysis with the large subunit counterparts and *in silico* studies on their promoter sequences, gene structures, protein properties and phylogeny. These observations are reported here.

## Materials and methods

### RPS gene sequence retrieval

A list of all ribosomal protein family (both large and small subunit) was obtained using a keyword search, “ribosomal” in the RGAP-DB^1^ and Phytozome v11^2^ databases. From the list, a total of 56 ribosomal small subunit genes (40S) were shortlisted by their names starting with the letter “S” as a prefix (S for Small subunit). Nucleotide sequences of all the 56 genes were retrieved from RGAP-DB. These 56 sequences were further confirmed for their ribosomal origin through nucleotide and protein BLAST^3^ search in the NCBI^4^ and Hidden Markov Model (HMM) of Pfam^5^ databases, respectively. The availability of only 34 ribosomal small subunit proteins, which are coded by 56 genes indicated the existence of paralogs of the genes coding for more than one RPS protein. For the gene expression analysis, we selected 34 RPS genes, one from each group representing their corresponding paralogous groups. Identical gene copies were, thus, excluded from further analyses. We have listed the 34 genes along with their paralogs in the Supplementary Table 9. The sequences of RPS genes were retrieved from RGAP-DB (a Nipponbare database). Our analysis of gene expression is performed in an *indica* rice variety (Samba Mahsuri). Hence, we cross-checked the sequence similarity of the coding as well as the 5′- upstream regions of the genes selected for analysis in the present investigation in both *indica* and *japonica* sequence databases by doing a detailed BLAST analysis of the *japonica* sequences on *indica* genome databases, OryGeneDB^6^ and EnsemblPlants^7^. Further, we have looked for the similarity of rice (40S) RPS genes with RPS genes of *Arabidopsis* and performed a BLAST search of the Rice sequences in TAIR^8^ database and listed the similarity percentage in Table 1.

**Table 1:** Similarity of Ribosomal protein small subunit (RPS) genes in rice with that in *Arabidopsis*.

**S. No**	**Ribosomal Protein Small Subunit (RPS) genes in Rice**	**RPS genes in *Arabidopsis***	**% similarity in amino acid sequence between Rice and *Arabidopsis***
1.	S3a LOC_Os02g18550	S3Ae AT4G34670.1	79
2.	S4 LOC_Os01g25610	Ribosomal S4 Family protein AT5G58420.1	83
3.	S4a LOC_Os05g30530	• Ribosomal S4 protein family AT5G58420.1 • Ribosomal protein S4 family (RPS4A) AT2G17360.1	82 81
4.	RPS5 LOC_Os01g01060	Ribosomal protein 5A AT3G11940.2	80
5.	RPS5A LOC_Os11g29190	• Ribosomal protein 5B AT2G37270 • Ribosomal protein 5A AT3G11940	78 80
6.	RPS6 LOC_Os01g12090	Ribosomal protein S6 family AT3G18760	89
7.	RPS6a LOC_Os03g27260	Ribosomal protein S6e RPS6B AT5G10360	82
8.	RPS7 LOC_Os02g21900	Ribosomal protein S7e family protein AT1G48830	85
9.	RPS7a LOC_Os03g18570	• Ribosomal protein S7e family protein AT1G48830 • Ribosomal protein S7e family protein AT3G02560	84 78
10.	RPS9 LOC_Os07g43510	Ribosomal protein S4 AT5G15200	81
11.	RPS9-2 LOC_Os03g05980	• Ribosomal protein S4 AT5G15200 • Ribosomal protein S4 AT5G39850	79 79
12.	RPS10 LOC_Os01g73160	S10 domain containing protein AT4G25740	92
13.	RPS10a LOC_Os02g34460	S10 domain containing protein AT5G52650	92
14.	RPS13 LOC_Os07g38540	Ribosomal protein 13a A AT4G00100	87
15.	RPS13a LOC_Os08g02400	• Ribosomal protein S13a AT4G00100 • Ribosomal protein S13 AT3G60770	84 82
16.	RPS15 LOC_Os07g08660	Cytosolic Ribosomal protein S15 AT1G04270	87
17.	RPS15a LOC_Os02g27760	Ribosomal protein S15A AT1G07770	81
18.	RPS17 LOC_Os03g01900	Ribosomal protein S17 family protein AT3G10610	83
19.	RPS18 LOC_Os03g58050	Ribosomal protein S18 family AT1G22780	83
20.	RPS18a LOC_Os07g07719	Ribosomal protein S18 protein family AT1G34030	81
21.	RPS18b LOC_Os07g07770	Ribosomal protein S18 (RPS18C) AT4G09800	82
22.	RPS19 LOC_Os03g31090	Ribosomal protein S19e family protein AT5G61170	80
23.	RPS2 LOC_Os03g46490	Ribosomal protein S21e AT3G53890	84
24.	RPS23 LOC_Os03g60400	Ribosomal protein S23family protein AT5G02960	83
25.	RPS23a LOC_Os10g20910	• Ribosomal protein S23 AT5602960 • Ribosomal protein S23 AT3G09680	81 82
26.	RPS24 LOC_Os01g52490	Ribosomal protein S24e family protein AT5G28060	82
27.	RPS25 LOC_Os08g44480	Ribosomal protein S25 family protein AT4G39200	85
28.	RPS25a LOC_Os11g05562	• Ribosomal protein S25 family protein AT4G39200 • Ribosomal protein S25 family protein AT2G21580	87 84
29.	RPS26 LOC_Os01g60790	Ribosomal protein S26e family protein AT2G40590	87
30.	RPS27 LOC_Os04g27860	Ribosomal protein S27 AT3G61110	83
31.	RPS27a LOC_Os01g22490	Ribosomal protein S27a AT1G23410	81
32.	RPS28 LOC_Os10g27174	Ribosomal protein S28 AT5G64140	95
33.	RPS29 LOC_Os03g56241	Ribosomal protein S29e family protein AT4G33865	85
34.	RPS30 LOC_Os02g56014	Ribosomal protein S30 family protein AT2G19750	83

*Similarity percentage of rice ribosomal protein small subunit (RPS) genes in rice with the corresponding RPS gene in Arabidopsis has been mentioned after a BLAST analysis in TAIR database*.

### Chromosomal location of RPS genes

**Figure 1:**
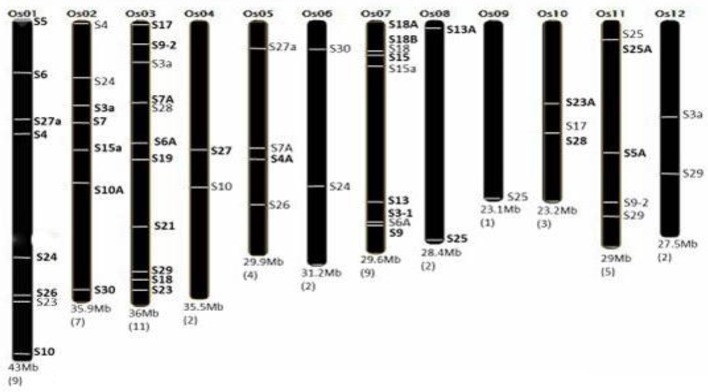
Distribution of RPS genes in rice genome. Number of RPS genes corresponding to the particular chromosome is mentioned in bracket below. The RPS genes and their location on the particular chromosome are marked accordingly. The chromosome number and size are also mentioned. This figure is adapted and modified from Moin et al. (2016b), under CC BY 4.0 license.

To obtain the genome-wide chromosomal distribution of all RPS genes on 12 chromosomes of rice, we had submitted the locus numbers (obtained from RGAP-DB) of 56 RPS genes as an input to the OrygenesDB. Based on the OrygenesDB outputs, all RPS gene locations were indicated on the chromosome maps, and the number of genes on each chromosome has also been shown. Genes, which were selected for expression analysis, have been shown in bold in Figure 1.

### Structural features of RPS genes

To predict the gene structure features, such as intron and exon number, size, etc., we had submitted the genomic and corresponding cDNA sequences of the selected 34 genes to the Gene Structure Display Server (GSDSv2)^9^.

### Putative promoter survey of 34 RPS genes

Since all the 34 genes exhibited differential expression under multiple stress treatments, we checked for the presence of putative *cis-*elements in the putative promoter regions of the corresponding genes. To verify this, we have used an *in silico* approach, in which the nucleotide sequence of about ≤ 1 kb upstream of each of the 34 RPS genes was retrieved from RGAP-DB, and the sequences were cross-checked for their identity/ deviation with the *indica* genome in OrygeneDB and EnsemblPlants databases. Further, the sequences that exhibited the highest similarity with *indica* genome in the databases were submitted to PlantCARE (Cis-Acting Regulatory Elements) Database^10^ (Rombauts et al., 1999; Dhadi et al., 2009; Ding et al., 2011) for identifying the regulatory elements. We have compared the putative promoter regions of some of the significant genes in two other databases, such as PlantPAN 2.0^11^ and the JASPAR^12^ and found the functional similarity of the elements with respect to PlantCare database confirming the authenticity of our observations.

### Ribosomal protein structural and phylogenic properties

Predicted protein sequences available from the RGAP-DB were used for predicting the protein structure, molecular weight, isoelectric point (*p*I) prediction *in silico* by the online tool PSORT^13^ and ExPASyProtParam^14^. By submitting the protein sequences to SMART^15^ (Simple Modular Architecture Research Tool), we have identified various ribosomal domains and motifs. Sequence submission to ExPASyProtParam helped us in obtaining the GRAVY indices of RPSs, which indicated the probable hydrophobicity of the proteins by their negative values for the indices that are relevant for the hydrophobic proteins.

To predict the three-dimensional structure and to check for the presence of ligand binding sites on each RPS, we have used the Phyre2^16^ (Protein Homology/AnalogY Recognition Engine v2; Kelley et al., 2015) and 3DLigandSite^17^ programs (Wass et al., 2010), respectively. Results from these tools predicted the protein properties like percentage of α-helix, β-sheet, disordered protein fraction and ligands of each protein, ligand binding residues on the proteins and 3D model of the ligand bound protein. Multiple sequence alignment using ClustalW^18^ also deciphered the phylogenetic relationships among the 34 RPS proteins in MEGA6^19^ (Molecular Evolutionary Genetic Analysis) platform, which constructed an unrooted phylogenetic tree showing evolutionary sequence similarity among the proteins with bootstrap values of 100.

### Plant material, growth conditions and stress treatments

*Oryza sativa* L. ssp. *indica* var. Samba Mahsuri (BPT-5204) seeds were surface sterilized using 70% ethanol for 50 s followed by washing with 4% sodium hypochlorite for 15–20 min and 5–6 washes with sterile double-distilled water. After this, the seeds were dried on sterile blotting paper and were placed on solid MS media at 28 ± 2°C and 16 h light/8 h dark photoperiod cycle (Moin et al., 2016b).

For native expression studies, the surface sterilized seeds were allowed to germinate on solid MS medium. Some of the seeds were soaked for 16 h to collect the embryo and endosperm tissues using a sterile surgical blade. Shoot and root apical meristems of 3 d old seedlings and roots and shoots of 7 d old seedlings were collected for the expression studies. After transferring the seedlings from MS medium to soil in the pots; roots, shoots, leaves, root-shoot transition region, flowers, grains and panicles were collected (Moin et al., 2016b).

For abiotic stress treatments, 7 d old seedlings were exposed to ABA (100 μM), PEG (10%), NaCl (250 μM), and H_2_O_2_ (10 μM) by dipping them in the respective solutions. Around five seedlings in replicates were collected after 15 min, 3, 6, 12, 24, 48, and 60 h duration. Roots and shoots were collected separately for RNA isolation and cDNA synthesis. The untreated seedlings in water were used as control samples for normalization in qRT-PCR (Moin et al., 2016b).

To analyse the expression of the RPS genes under biotic stress, rice samples infected with *Xanthomonas oryzae* pv. *oryzae* (*Xoo*) and *Rhizoctonia solani* were collected 20 and 25 d post infection, respectively. For the biotic stress experiments, 1-month-old rice plants were used for treatments. The untreated samples of the same age were used as corresponding controls. The suspension of *Xoo* bacterium was applied at the edge of the leaves using a sterile blade (Moin et al., 2016b) and agar blocks of *Rhizoctonia solani* were placed on the leaf sheaths (Park et al., 2008) as per the standard protocols (and maintained under controlled culture room conditions.

All the samples were collected as three biological and technical replicates, and qRT-PCR was performed with their respective control samples. Rice specific *act1* and β-*tub* genes were used as internal reference genes for normalization and the mean values of relative fold change were calculated as per ΔΔC_T_ method (Livak and Schmittgen, 2001). For native expression studies, the normalization was performed with only rice specific *act1* and β-*tub* genes (ΔC_T_). Bar diagrams and heat maps were generated by Sigmaplotv11 and Morpheus^20^ programs, respectively using the means of the fold change that were calculated from the qRT-PCR data.

### RNA isolation, cDNA synthesis and quantitative real-time PCR (qRT-PCR)

Total RNA was isolated from the treated and untreated samples using Tri-Reagent (Takara Bio, UK) according to the manufacturer's protocol. The concentration of the isolated RNA was checked using a Nanodrop spectrophotometer, and 2 ug of RNA was used to synthesize first strand cDNA using reverse transcriptase (Takara Bio, UK). The synthesized cDNA was diluted ten times, and 2 μl of it was used to perform qRT-PCR and 10 μM of the primers for each gene was used per reaction. The primers specific to RPS genes were designed by using the on line tool Primer-3^21^ the sequences of forward and reverse primers are listed in Supplementary Table 5. The amplification protocol included an initial denaturation at 94°C for 2 min, with 40 cycles of 94°C for 30 s, primer specific annealing temperature for 25 s, an extension of 72°C for 30 s. This was followed by constructing a melt curve at the end to estimate the amplification efficiency of each gene. The fold change was calculated as per ΔΔ*C*_T_ method, and the mean of these values was considered as the final fold change of the transcript levels (Moin et al., 2016b). Means of relative fold change were plotted in Sigmaplot v11 with standard error to produce the bar diagrams for all the genes in a specific stress.

## Results

### Chromosomal distribution

Rice genome has 12 chromosomes carrying 56 RPS genes. Based upon the OrygeneDB output, the RPS genes were found to be distributed throughout the rice genome covering all 12 chromosomes. Both the arms of chromosome randomly carried the RPS genes. Each chromosome carried at least one member of the RPS gene family. Most of the genes are located on chromosomes 3, 1, 7, and 11, with chromosome 3 bearing the highest number of eleven RPS genes. Both the chromosomes 1 and 7 carried a total of eight and nine genes, respectively. Chromosome 9 is the only example with only one RPS gene present near the telomere region of its long arm. The chromosome 10 and 1 carried three and eight RPS genes, respectively (Figure 1).

### Gene structure prediction

RPS genes, like other eukaryotic genes, showed the organization of introns, exons and UTRs. On submitting the nucleotide sequences of the 34 RPS genes in the Gene Structure Display Server (GSDS), we have obtained the predicted gene structure showing all exons, introns, UTRs for each gene and the number and relative position of each of the components. The BLAST analysis of RGAP-DB sequences with *indica* variety database showed that the coding regions of the 34 genes in both the varieties were similar. The percent similarity of the coding and the 5′-upstream regions of the genes found from the BLAST analysis has been listed in Supplementary Table 1. The similarity of RPS genes in rice with the corresponding RPS gene in *Arabidopsis* has also been searched with the help of BLAST analysis in TAIR database. All the 34 RPS genes studied in this report exhibited very high similarity with their counterparts in *Arabidopsis* also. The minimum similarity percentage is 78 and the maximum is 95.

Results from the GSDS suggested that most of the RPS genes consisted of both introns and exons (Supplementary Figure 1). *RPS25a* has a long stretch of intronic sequence covering most of its length. It is also the longest gene among the selected genes with a total length of approximately 6 kb. *RPS17* and *RPS30* lacked introns and possessed a single and small CDS flanked by UTRs. *RPS6, RPS19, RPS23a*, and *RPS7* lacked UTRs and are only composed of intronic and exonic sequences. *RPS23a* is the smallest gene with a total size of 0.5 kb. The number of exons also varied from a minimum of two in *RPS23a* and *RPS29* to a maximum of three exons in *RPS3a*. The details of properties related to the gene structure have been provided in Supplementary Table 2.

### Putative promoter analysis

In accordance with the observations regarding the stress responsive differential expression and tissue specific expression of several RPS genes, we tried to correlate the transcript levels with the presence of stress-responsive elements in the 5′ upstream regions of the individual genes. Among the 5′ regions of the RPS genes, we have shortlisted some of the common stress-responsive elements that were present in single or multiple copies, and we have listed the elements that might have significance in tissue specificity of expression in Supplementary Table 7. Most of the genes have elements for meristem and endosperm specificity in their promoter regions.

**Figure 2:**
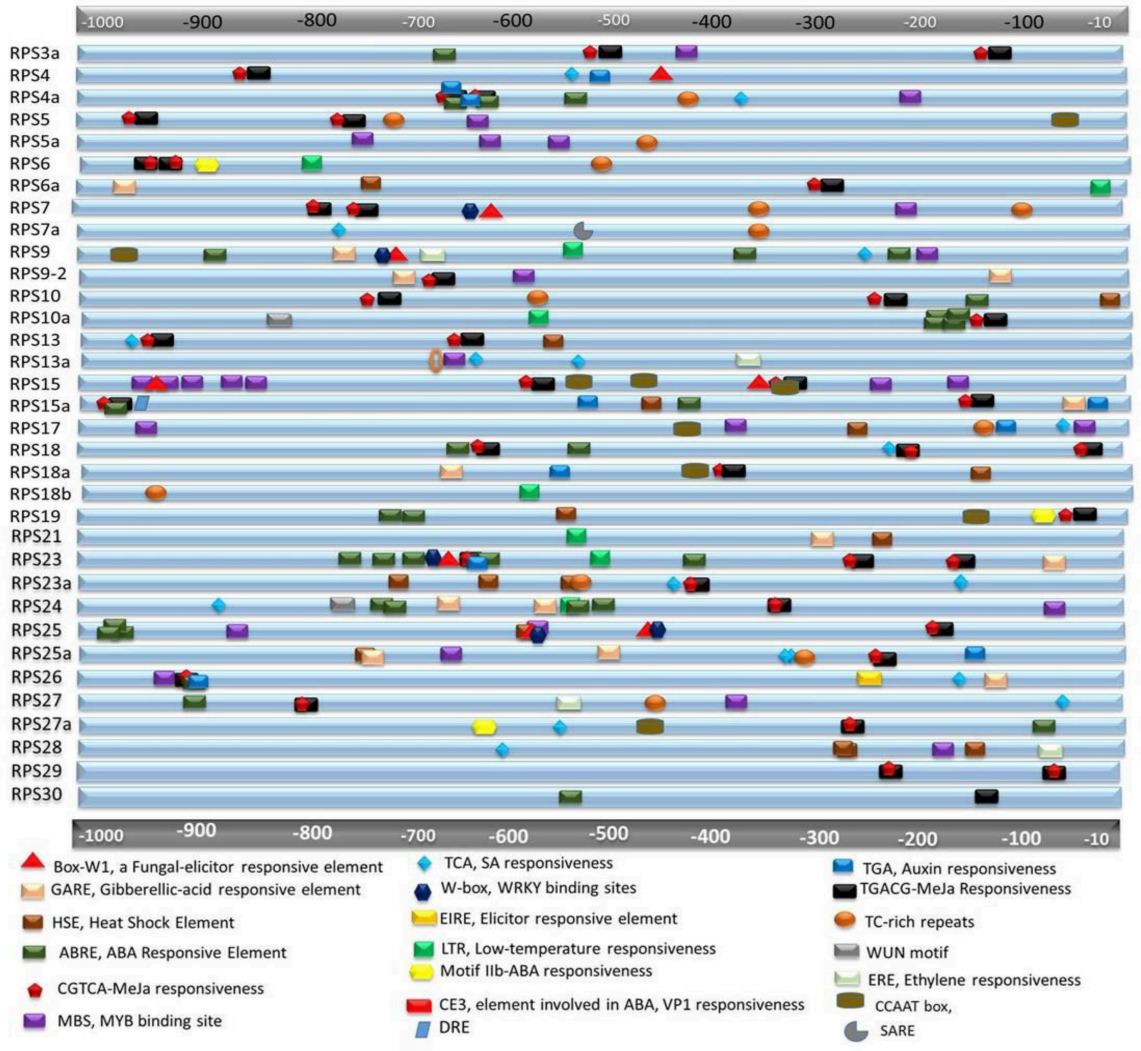
*In silico* analysis for the presence of *cis*-regulatory elements in the putative promoter regions of RPS genes. About ≤ 1 kb upstream region of each gene is retrieved and searched for *cis-*regulatory elements that might be responsible for stress responsiveness. Different elements have been demonstrated in different shapes and colors and they are approximately placed according to their actual location in the genome. The functions of each of the elements are mentioned below the figure.

All the RPS genes have at least two stress responsive elements in their immediate upstream regions (up to 1 kb). *RPS30* has one copy of ABRE and TGACG, and *RPS18b* has one copy of LTR and TC rich repeat as their *cis*-acting regulatory elements (CAREs). Other than these two genes, putative promoter regions of the rest of the genes have more than two elements. SARE, EIRE, motifIIb, DRE and ERE, which are responsive to SA, elicitor, ABA, dehydration and ethylene, respectively and are present in single copies in the putative promoter regions of some of the genes. *RPS23* showed five repeats of ABREs, three copies of TGACG motif, three copies of CGTCA motif and one copy each of TGA, Box W1, W box, and GARE elements. *RPS4a* exhibited three copies of ABREs, two repeats each of TGA, TGACG, and CGTCA elements. *RPS15* has two repeats of MBS. Similarly, the genes *(RPS4, 10a, 6a*, and *5*) that have exhibited remarkable up-regulation under biotic stress conditions have multiple MeJa responsive elements and some of them contain box W1 and TCA elements that are related to responsiveness under pathogen attack. All the stress responsive *cis*-acting elements were pictorially represented by their physical mapping in Figure 2 and Table 2 mentioning the function of the corresponding motifs. The elements having roles in tissue specificity for each of the genes have been listed in Supplementary Table 7. The BLAST analysis for the 5′-upstream regions of the genes of both the varieties showed that 8 out of 34 genes had shown a slight variation in the sequences of upstream regions (97%) in the two varieties. The differences in regulatory elements due to the dissimilarity in their sequences have been compared and shown in the Supplementary Figure 7. The upstream regions could not be identified for three genes (*S15a, S18a*, and *S18b*) for which the promoter analysis has been performed using the *japonica* sequences only.

**Table 2:** Functions of different stress responsive elements found in the promoter regions of the RPS genes.

**Name of the elements**	**Function**
ABRE (ABA responsive elements) DRE (dehydration responsive elements)	Osmotic stress-mediated transcriptional regulation (Kim et al., 2011).
HSE (heat stress element)	Tolerance to heat stress.
LTR (low temperature responsive)	Cold responsive.
MBS (MYB binding site)	Drought induced expression of genes involved in defense (Sazegari et al., 2015).
W-box- binding site of WRKY transcription factors	Pathogen induced response (Rushton et al., 2002).
Box-W1-fungal-elicitor responsive element	Involved in defense signaling (Hernandez-Garcia and Finer, 2014).
TCA and SARE	Responsive to salicylic acid.
TGACG and CGTCA	Responses related to methyl jasmonate.
ERE (ethylene responsive elements)	Crucial for stress signaling.
TGA and AuxR	Auxin responsive (Sutoh and Yamauchi, 2003; Bastian et al., 2010; Sazegari et al., 2015).
motif IIb and CE3 (coupling element 3)	Involved in ABA mediated osmotic stress signaling (Hobo et al., 1999).
WUN motif (wound specific)	Involved in wound response (Pandey et al., 2015).

### Protein properties and phylogeny

The submission of the protein sequences from RGAP-DB to the ExPasyProtParam server revealed many putative properties. Their molecular weights (MW) varied from approximately 7 to 30 kDa with RPS4 having the highest MW of 29.8 kDa and RPS30 with the lowest MW of 6.9 kDa. In accordance with the MW, the RPS4 is the largest protein with a sequence of 265 amino acids, while RPS30 is the smallest with 62 amino acids. *In silico* analysis using ExPasyProtParam server helped us to get the theoretical *pI*-values of each of the RPS. Most of them were in the range of 9 to 12 with very few exceptions out of this range. For example, RPS6 has a very low *p*I of 4.86. Most of the RPS proteins are rich in positively charged amino acids like Arg and Lys compared to negatively charged amino acids like Asp or Glu. The only exceptions are the RPS21, which has an equal number of both the types and RPS6 having more of acidic amino acids. Percentage composition of secondary protein structures (disordered protein, alpha helix and beta sheet) has variations within the RPS family. RPS4 has the minimum value of 14% while the RPS30 has the maximum value of 76%, respectively; S10a exhibited 10%, and S29 and S13 have 64% α- helical content, respectively. The range on the percentage of β-sheet content also varied with RPS13, 13a having 0% and S27 showing 49% of the β-sheet content.

The Grand Average of Hydrophobicity (GRAVY) indices were below the value of zero for all 34 RPS proteins suggesting the hydrophobic nature of RPS proteins. We have also identified some of the interacting ligands of these RPS proteins from the 3DLigandSite. The metal and non-metal ligands include IF-1, FMN, FAD, Mg^2+^, Zn^2+^ etc. and the specific residues involved in the ligand binding have been listed in the Supplementary Table 3. Most of the RPS proteins have Low Complexity Region (LCR) motifs, which are the stretches of biased amino acid sequences having a long repetition of single or few amino acids and they have been connected to many protein functions and interactions. LCRs that are located terminally in a protein have been considered to be translationally enriched and have stress responsive roles (Coletta et al., 2010). Three dimension protein structures from 3D ligand site have been included in Supplementary Figure 2. The ribosomal protein sequences were submitted to SMART database, and it was found that all of them exhibited ribosomal domains. Some of the proteins also contain KOW domain (for e.g., *RPS4, RPS4a*). *RPS6* has a SPT2 domain; *RPS5a* has a HLH domain, a CARD domain and an ANTAR domain. *RPS18a* has a SANT domain, which is a MYB- related domain that binds to DNA and helps in transcriptional regulation (Feller et al., 2011) and it also has a homeobox domain HALZ. All the domains associated with each of the RPS subunits are listed in Supplementary Table 8.

Protein sequence data was exploited to construct an unrooted phylogenetic tree of 34 RPS proteins in the MEGA6 software. Alignment with ClustalW employing a neighbor joining algorithm resulted in the generation of a tree that showed the evolutionary relationship between the RPS proteins. The tree suggested that a considerable number of cladogenesis events occurred during the course of evolution in the RPS gene family that was reflected in their protein sequence similarity and diversity. The earliest divergence separated the proteins into two major clades, of which one contains five proteins (RPS13, 13a, 3a, 19, and 21) and the other major clade carried all the other proteins, which has further diverged several times. These five RPS genes were checked for similarity with their paralogs and depicted as a phylogenetic tree in Supplementary Figure 3B. While *RPS 19* and *21* have no paralogous members, *RPS13* and *RPS3a* have two paralogous members, and they have shown similarity with their corresponding group. Nodes, from where some pairs of genes were diversified showed the bootstrap value of 100. Such high value indicates the high significance of the clade. The phylogenetic tree showing the evolutionary relationships among the RPSs is shown in Supplementary Figure 3.

### Native expression profiling of the RPS genes in different tissues

**Figure 3:**
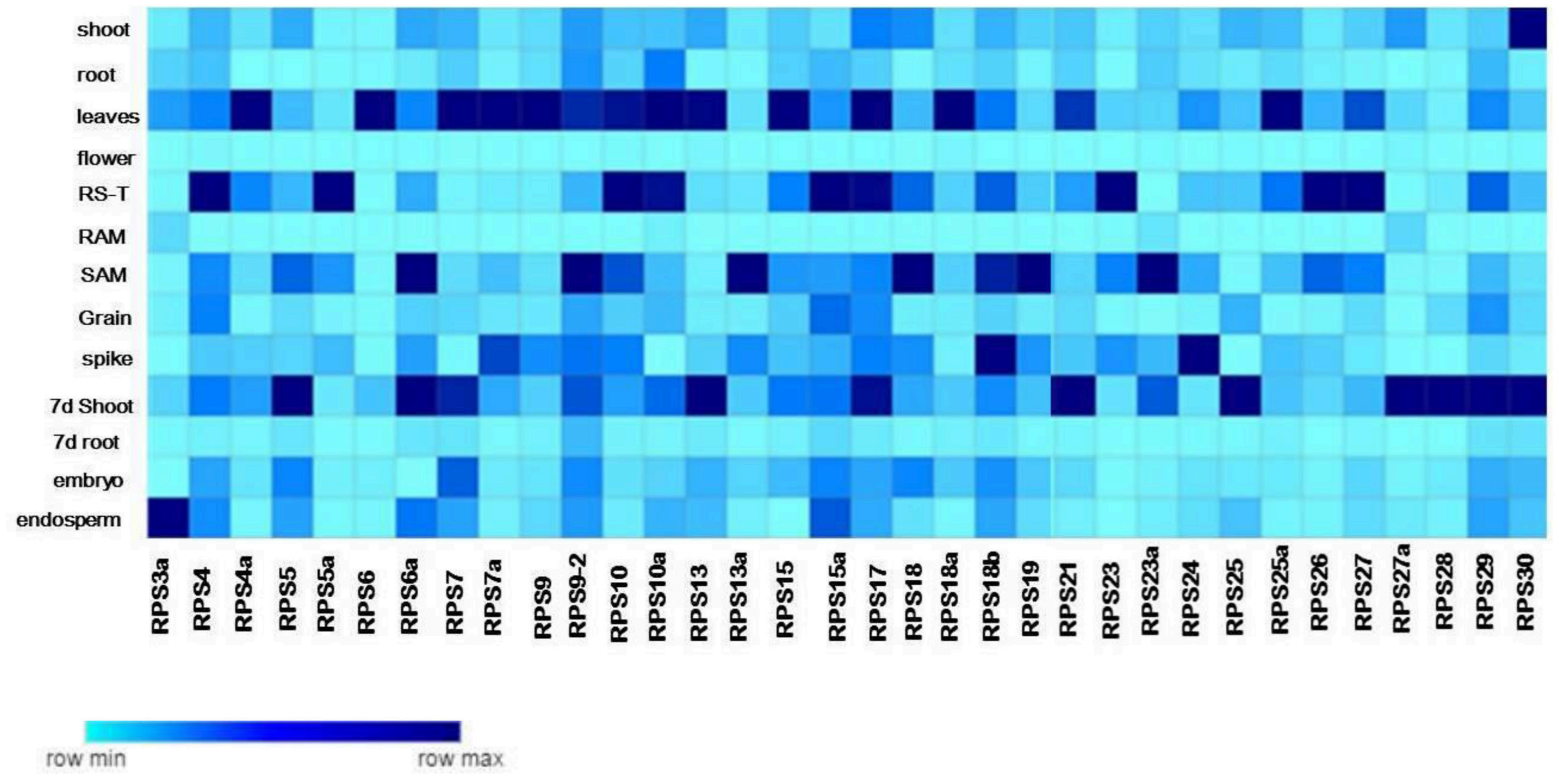
Tissue specific expression of the RPS genes. The native expression of 34 RPS genes in 13 tissues is determined by qRT-PCR with single normalization with rice actin. The mean values of the fold change of the biological and technical replicates are represented in the form of a heat map.

To gain insights into the expression patterns of RPS genes in rice development and also to provide a comparative analysis between RPs of both the large and small subunits, we performed qRT PCR of 34 RPS genes in 13 different tissues as described in Moin et al. (2016a) for the RPL genes. The mean values were utilized to construct heat maps to demonstrate clearly the native expression pattern of the RPS genes (Figure 3). The tissues used to study the native expression pattern included embryo and endosperm from 16 h old germinating seed, plumule and radicle from 3 d old seedlings; shoot and root of 7 d old seedling; shoot, root, flag leaves, panicle, flower, grains and root-shoot transition of mature 20 d old plants (after transfer to green house).

The complete tissue-specific up-regulation pattern of the RPS genes in native conditions has been summarized in Supplementary Table 4 and also in the form of heat maps (Figure 3). The up-regulation of RPS genes was significant in mature leaves (25 genes), shoots (21 genes), 7 d shoots (26 genes), plumules (21 genes), and root-shoot transition (23 genes). Apart from these four, seven and 15 genes were up-regulated in seedling radicles, 7 d old root and mature roots, respectively. An analogous pattern of gene expression was also observed under different stress conditions as well, where more number of RPS genes was found to be up-regulated in shoot than in the root tissues showing that the majority of the RPS genes were up-regulated in shoots, leaves and plumules compared to the other organs.

About 17 RPS genes showed expression in rice grains and panicles indicating their involvement in inflorescence development and grain filling. None of the genes was expressed in florets implying that RPS proteins have probably no significant roles in the development of this meiotically active tissue. About 18 and 15 RPS genes were up-regulated in embryo and endosperm, respectively showing that these genes might have a role in seed development and maturity. *RPS23a, RPS27a, RPS10a*, and *RPS7* have exhibited no significant up-regulation in any of the tissues and *RPS23* is up-regulated only in root-shoot transition. A considerable up-regulation of *RPS28* and *RPS29* was observed in roots under different stresses. *RPS4, RPS5a, RPS7a, RPS9, RPS9-2, RPS13, RPS6, RPS17, RPS18b, RPS24*, and *RPS29* were up-regulated in most of the tissues, while *RPS13a, RPS18a, RPS4* were up-regulated in shoots throughout the duration of all the stress conditions. Similarly, these genes were also expressive in shoots, leaves, plumules and in native conditions as well.

### Differential expression analysis of the RPS genes in response to abiotic stress treatments

Our findings on the screening of a large-pool of activation tagged mutant population for high water-use efficiency revealed the significant up-regulation of RPL genes (*RPL6* and *RPL23A*) by the integrated enhancers in two of the mutant plants with sustained productivity under limited water conditions (Moin et al., 2016a). In the follow-up study, an extensive tissue specific and differential expression study of the entire RPL gene family was carried out, which showed significant up-regulation of certain RPL genes under abiotic and biotic stresses (Moin et al., 2016b). Results from these two studies prompted us to further investigate the expression of 40S Ribosomal Protein genes in response to abiotic and biotic stress conditions and to arrive at proper combinations of RPS and RPL genes for subsequent genetic manipulation. For studying their differential expression, we have selected 34 RPS candidate genes from the respective paralogous groups in response to four abiotic stresses (ABA, PEG, NaCl, and H_2_O_2_) at seven time points and two biotic stresses (BLB and SB). ABA, PEG, NaCl, and H_2_O_2_ induce a stress similar to osmotic, drought, salinity and oxidative stress, respectively (Gao et al., 2007; Hamayun et al., 2010; Hossain et al., 2015; Sah et al., 2016). Treatment of the plants with these stress-inducing agents would mimic the corresponding stress situations and consequently activate numerous stress responsive pathways. Also, we also analyzed their native expression pattern in different tissues.

The genes that exhibited ≥ 3 fold transcript level on the log_2_ scale were considered as up-regulated. The pattern of expression of the genes depended mostly on the type of stress signal and also the tissue in which the expression was studied. We have categorized the genes in terms of their timing and the intensity of response. Some of the genes were up-regulated as early as 15 min-3 h after the onset of the stress, while a few of them responded between 3 and 12 h after exposure while others were up-regulated 12 h after the application of the stress. We have differentiated them as Immediate Early (IE), Early (E), and Late (L) expressing genes, respectively.

Since the level of expression varied from 3 to several 100 folds, we have further categorized the up-regulated genes as highly expressive genes (≥30 fold), moderately expressive (between 10 and 30 fold) and low-expressive (between 3 and 10 fold). The expression of the RPS genes in the shoot samples has been observed to be higher compared to the corresponding root samples under almost all the abiotic stresses at different time intervals. Under some conditions, the up-regulation of the genes in the shoot samples has been detected to be several 100 folds, which is not the case for the corresponding root samples. In the case of shoot samples, all the genes showed significant up-regulation for all the stress conditions for at least one time point, and they were predominantly IE type in their response, except under H_2_O_2_ treatment.

Although all the genes were up-regulated under all the stress conditions in shoots, their mode of response varied with the nature of the stress. For example, ABA induced the up-regulation of 26% of the genes *(RPS7, 15a, 18a, 25a, 4, 7a, 10, 10a*, and *13a*) and these maintained a significant expression (≥3-fold) until the last time point used (60 h). Of these, 50% were highly expressive (*RPS7, 15a, S8a*, and *25a*) with >30-fold transcript level throughout the stress treatment up to 60 h time point. *RPS7* and *RPS18a* showed up-regulation up to several 100 folds. PEG and NaCl also induced the up-regulation of a majority of genes in shoots until 60 h time point, except *RPS7a* and *RPS23* and *RPS5, RPS10a*, and *RPS23a* under PEG and NaCl treatments, respectively, which became down-regulated.

**Figure 4:**
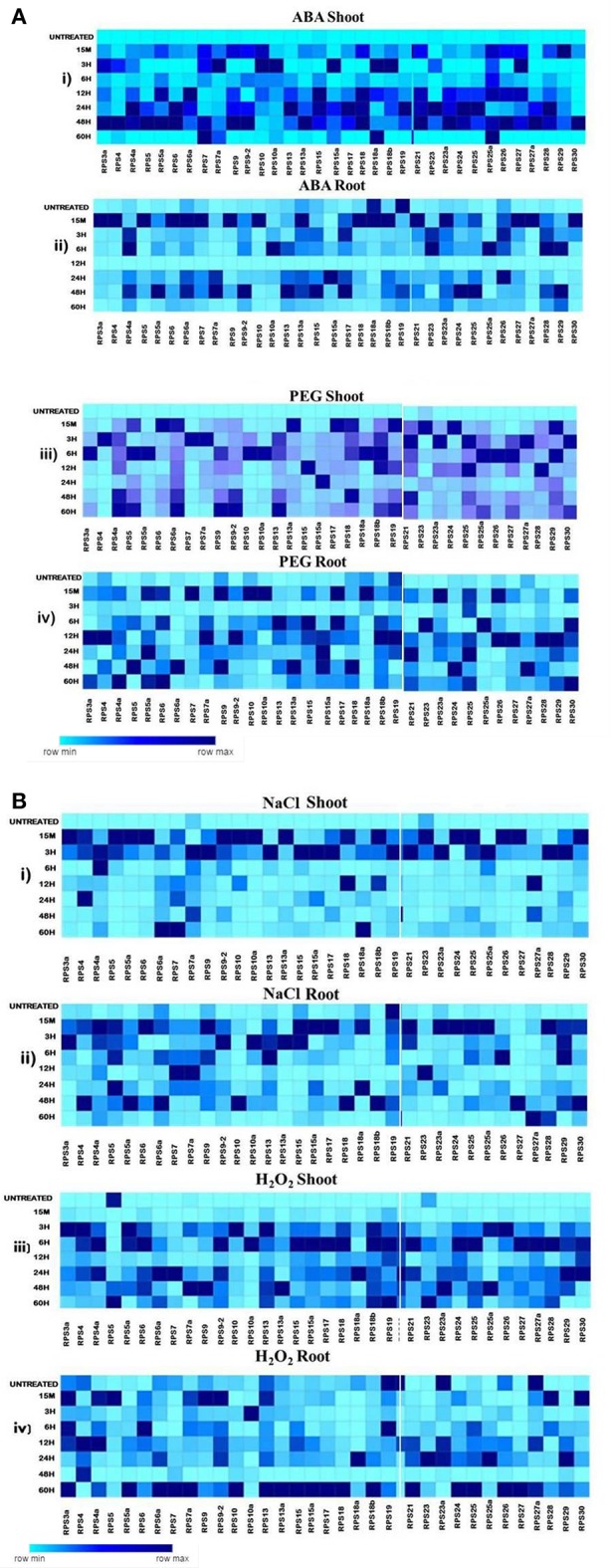
Heat map representation of RPS genes in response to ABA, PEG, NaCl and H_2_O_2_ treatments. The 7d old seedlings were treated with ABA(100 μM), PEG (10%), NaCl (250 μM), and H_2_O_2_(10 μM) and qRT-PCR is used to determine the level of expression of 34 RPS genes in the shoot and the root tissues at the time points mentioned on the left side. The fold change has been normalized by ΔΔC_*T*_ method with respect to the unstressed seedlings maintained in water. Rice specific *act1* and β-*tub* genes were used as endogenous reference genes. The mean values of the fold changes of the biological and technical replicates were considered for preparing the heat maps. **A**-(i), (ii), (iii), and (iv) represent the expression of the genes in shoot and root tissues under ABA and PEG respectively and **B**- (i), (ii), (iii), and (iv) represent the expression of the genes in shoot and root tissues under NaCl and H_2_O_2_ respectively. The darkest color depicts the highly up-regulated genes whereas the lightest color represents the less up-regulated and down-regulated genes.

Some of the RPS genes maintained remarkably high transcript levels (≥100 fold). These include *RPS4a, RPS9, RPS9-2, RPS13, RPS17, RPS19, RPS27*, and RP*S30* under PEG treatment and *RPS4a, RPS7, RPS9-2, RPS18a*, and *RPS23a* under NaCl treatment. The up-regulation of *RPS4, RPS7, RPS9, RPS10, RPS19*, and RP*S26* under salt treatment is in agreement with the earlier studies (Kawasaki et al., 2001). Unlike in ABA, PEG, and NaCl treatments, several RPS genes were late responding in shoots under H_2_O_2_ treatment. Moreover, the level of up-regulation was also not as high as in other treatments. About 60% of the genes were up-regulated 15 min post treatment. About 30 genes have been found to maintain a considerable level of expression, and among these, *RPS4a, RPS9-2, RPS15a, RPS18*, and *RPS28* have exhibited ≥100 fold up-regulation. In roots, *RPS19* was down-regulated persistently in response to all the abiotic stresses at all the time intervals. *RPS10a, RPS18, RPS18a, RPS18b, RPS24, RPS27a*, and *RPS6a* were also observed to maintain a very low transcript level. Some of the genes that were initially up-regulated, subsequently, became down-regulated with the progression of the stress treatment. The expression pattern of all the genes under different stress treatments in the shoot and root tissues has been represented in the form of heat maps (Figures 4A,B).

**Figure 5:**
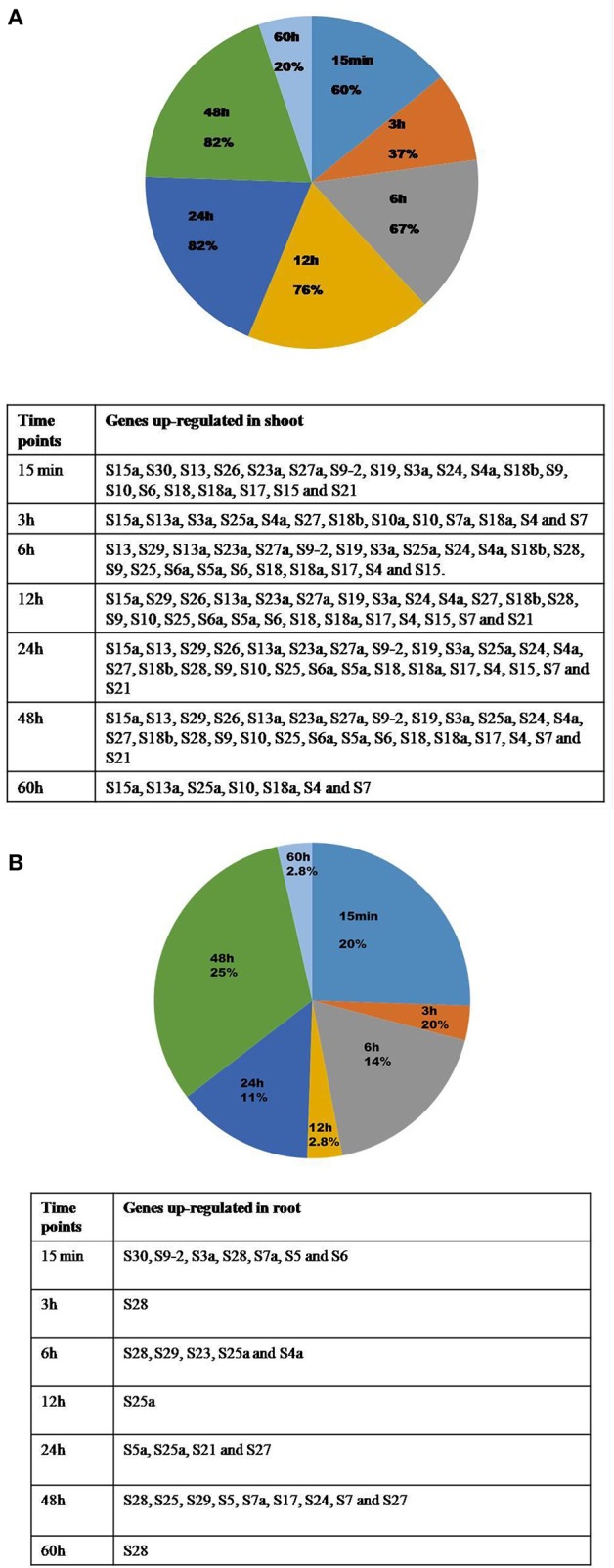
Percentages of genes up-regulated in shoot and root under all the stresses at different time points. The percentage of genes that are upregulated in shoot **(A)** and root **(B)** at different time intervals after the application of the stress is represented in form of pie charts with the genes enlisted in the respective tables.

There is an overlap in the expression of the genes that are up or down-regulated in response to the stresses at several time points, and this has been demonstrated in the form of Venn diagrams (Supplementary Figures 4, 5). Table 3 presents a detailed list of genes that were up/down-regulated in different stress conditions in the shoot and root tissues separately. The percentage of genes over-expressed at certain time point under the stress conditions in shoot and root has been represented in the form of Pie charts (Figures 5A,B), and the genes are tabulated in the tables that accompanied the figures. The genes that were commonly up-regulated under all the treatments, particularly during the early response may have significance in stress related signaling (Kilian et al., 2007) as the early responsive genes play a crucial role in defense against the environmental stresses (Mahajan and Tuteja, 2005; Ouyang et al., 2007).

**Table 3:** Regulation of 35 RPS genes in shoots and roots under 4 abiotic stresses.

**Locus Name**	**Protein**	**Regulation**	**Response**	**Maximum Fold Change**	**Regulation**	**Response**	**Maximum Fold Change**
			**Shoot**			**Root**	
**ABA**							
LOC_Os02g18550	RPS3a	UP	IE (47.23)	267.24	UP	IE (55.89)	55.89
LOC_Os01g25610	RPS4	UP	IE (10.22)	280.43	UP	IE (19.29)	19.29
LOC_Os05g30530	RPS4a	UP	IE (39.545)	197.20	UP	IE (6.06)	7.084
LOC_Os01g01060	RPS5	UP	IE (25.13)	114.8852	UP	IE (45.03)	45.035
LOC_Os11g29190	RPS5a	UP	IE (21.81)	55.41	UP	IE (7.235)	16.32
LOC_Os01g12090	RPS6	UP	IE (29.16)	140.87	UP	IE (29.55)	29.55
LOC_Os03g27260	RPS6a	UP	IE (31.25)	187.72	UP	IE (3.31)	3.315
LOC_Os02g21900	RPS7	UP	IE (132.89)	245.67	UP	IE (7.805)	7.805
LOC_Os03g18570	RPS7a	UP	IE (4.243)	12.568	UP	IE (6.48)	34.015
LOC_Os07g43510	RPS9	UP	IE (39.066)	58.97	UP	IE (21.89)	21.89
LOC_Os03g05980	RPS9-2	UP	IE (38.20)	73.30	UP	IE (4.59)	4.59
LOC_Os01g73160	RPS10	UP	IE (54.88)	71.79	UP	IE (14.77)	14.77
LOC_Os02g34460	RPS10a	UP	IE (5.646)	56.49	UP	E (9.458)	9.458
LOC_Os07g38540	RPS13	UP	IE (3.57)	76.67	UP	E (4.612)	6.664
LOC_Os08g02400	RPS13a	UP	IE (22.51)	90.80	DOWN	—	—
LOC_Os07g08660	RPS15	UP	IE (5.99)	28.99	UP	L (4.159)	4.159
LOC_Os02g27760	RPS15a	UP	IE (5.23)	224.545	UP	L (10.142)	10.142
LOC_Os03g01900	RPS17	UP	IE (35.00)	186.78	UP	IE (11.17)	17.22
LOC_Os03g58050	RPS18	UP	IE (44.34)	75.63	UP	IE (7.28)	7.28
LOC_Os07g07719	RPS18a	UP	IE (34.18)	278.54	DOWN	–	–
LOC_Os07g07770	RPS18b	UP	IE (54.40)	216.73)	UP	IE (6.34)	6.34
LOC_Os03g31090	RPS19	UP	IE (16.54)	153.23	DOWN	–	–
LOC_Os03g46490	RPS21	UP	IE (53.26)	105.17)	UP	IE (21.06)	21.06
LOC_Os03g60400	RPS23	UP	IE (11.64)	35.78	UP	IE (6.63)	7.728
LOC_Os10g20910	RPS23a	UP	IE (18.30)	114.56	UP	IE (5.55)	5.55
LOC_Os01g52490	RPS24	UP	IE (34.23)	382.71	UP	IE (3.43)	8.76
LOC_Os08g44480	RPS25	UP	IE (21.79)	106.15	UP	IE (14.5)	19.99
LOC_Os11g05562	RPS25a	UP	IE (41.42)	56.23	UP	IE (4.34)	58.42
LOC_Os01g60790	RPS26	UP	IE (16.23)	32.28	UP	L (3.121)	3.121
LOC_Os04g27860	RPS27	UP	IE (25.71)	49.96	UP	IE (26.53)	26.53
LOC_Os01g22490	RPS27a	UP	IE (6.57)	302.66	UP	IE (41.93)	41.93
LOC_Os10g27174	RPS28	UP	IE (61.53)	167.88	UP	IE(5.5)	9.73
LOC_Os03g56241	RPS29	UP	IE (128.60)	128.60	UP	IE (52.64)	213.33
LOC_Os02g56014	RPS30	UP	IE (23.244)	113.63	UP	IE (57.34)	57.34
**PEG**							
LOC_Os02g18550	RPS3a	UP	IE (26.5)	226.24	UP	IE (5.79)	13.56
LOC_Os01g25610	RPS4	UP	IE (16.58)	467.00	DOWN	–	–
LOC_Os05g30530	RPS4a	UP	IE (213.06)	641.1959	UP	IE (9.24)	15.29
LOC_Os01g01060	RPS5	UP	IE (36.84)	46.53	UP	IE (4.62)	47.36
LOC_Os11g29190	RPS5a	UP	IE (8.42)	140.56	UP	IE (5.07)	5.55
LOC_Os01g12090	RPS6	UP	IE (182.15)	182.15	UP	IE (19.90)	24.11
LOC_Os03g27260	RPS6a	UP	IE (61.71)	66.33	UP	L (12.40)	12.40
LOC_Os02g21900	RPS7	UP	IE (19.53)	531.87	UP	IE (18.66)	18.66
LOC_Os03g18570	RPS7a	UP	IE (3.14)	11.51	UP	IE (6.75)	36.64
LOC_Os07g43510	RPS9	UP	IE (32.99)	100.11	DOWN	–	–
LOC_Os03g05980	RPS9-2	UP	IE (364.83)	689.87	UP	IE (8.03)	14.98
LOC_Os01g73160	RPS10	UP	IE (14.67)	287.60	UP	IE (3.256)	3.256
LOC_Os02g34460	RPS10a	UP	IE (4.94)	72.60	UP	IE (11.54)	11.54
LOC_Os07g38540	RPS13	UP	IE (8.46)	96.86	UP	E (10.53)	10.53
LOC_Os08g02400	RPS13a	UP	IE (91.59)	91.59	DOWN	–	–
LOC_Os07g08660	RPS15	UP	IE (6.14)	142.01	UP	E (4.58)	4.58
LOC_Os02g27760	RPS15a	UP	IE (21.99)	333.14	UP	E (7.59)	8.97
LOC_Os03g01900	RPS17	UP	IE (413.73)	413.73	UP	IE (20.17)	20.17
LOC_Os03g58050	RPS18	UP	IE (134.83)	144.28	UP	L (3.07)	3.07
LOC_Os07g07719	RPS18a	UP	IE (34.17)	315.89	DOWN	–	–
LOC_Os07g07770	RPS18b	UP	IE (49.35)	91.722	UP	E (3.81)	4.90
LOC_Os03g31090	RPS19	UP	IE (202.27)	202.27	DOWN	–	–
LOC_Os03g46490	RPS21	UP	IE (36.49)	58.40	UP	IE (15.15)	29.51
LOC_Os03g60400	RPS23	UP	IE (6.05)	6.05	UP	IE (6.62)	33.39
LOC_Os10g20910	RPS23a	UP	IE (71.23)	218.64	UP	IE (7.91)	7.91
LOC_Os01g52490	RPS24	UP	IE (262.68)	262.68	UP	L (5.42)	5.42
LOC_Os08g44480	RPS25	UP	IE (26.27)	110.35	UP	IE (7.62)	9.43
LOC_Os11g05562	RPS25a	UP	IE (13.69)	41.51	UP	IE (26.88)	230.74
LOC_Os01g60790	RPS26	UP	IE (7.09)	70.88	UP	IE (3.29)	3.40
LOC_Os04g27860	RPS27	UP	IE (94.99)	131.19	UP	IE (9.57)	22.27
LOC_Os01g22490	RPS27a	UP	IE (38.79)	251.68	DOWN	–	–
LOC_Os10g27174	RPS28	UP	IE (110.33)	264.44	UP	IE (9.41)	32.11
LOC_Os03g56241	RPS29	UP	IE (33.12)	33.56	UP	IE (5.32)	146.67
LOC_Os02g56014	RPS30	UP	IE (33.43)	127.31	UP	IE (5.98)	9.07
**NaCl**							
LOC_Os02g18550	RPS3a	UP	IE (394.95)	394.95	UP	IE (10.16)	17.009
LOC_Os01g25610	RPS4	UP	IE (94.08)	107.34	UP	IE (4.21)	5.32
LOC_Os05g30530	RPS4a	UP	IE (209.01)	754.56	UP	IE (14.40)	14.40
LOC_Os01g01060	RPS5	UP	IE (64.99)	64.99	UP	IE (7.89)	8.65
LOC_Os11g29190	RPS5a	UP	IE (233.41)	233.41	UP	IE (16.07)	36.43
LOC_Os01g12090	RPS6	UP	IE (132.97)	132.97	UP	IE (5.44)	5.44
LOC_Os03g27260	RPS6a	UP	IE (7.03)	47.95	DOWN	–	–
LOC_Os02g21900	RPS7	UP	IE (87.15)	135.30	UP	IE (13.69)	32.01
LOC_Os03g18570	RPS7a	UP	IE (5.44)	5.44	UP		
LOC_Os07g43510	RPS9	UP	IE (167.46)	325.91	UP	IE (3.75)	3.75
LOC_Os03g05980	RPS9-2	UP	IE (1357.75)	1357.75	UP	IE (7.14)	11.27
LOC_Os01g73160	RPS10	UP	IE (380.49)	380.49	UP	L (8.40)	8.40
LOC_Os02g34460	RPS10a	UP	IE (129.63)	129.63	UP	IE (19.8)	19.8
LOC_Os07g38540	RPS13	UP	IE (25.66)	364.99	UP	IE (4.61)	7.37
LOC_Os08g02400	RPS13a	UP	IE (187.85)	187.85	UP	IE (3.52)	53.19
LOC_Os07g08660	RPS15	UP	IE (10.02)	88.46	UP	IE (3.18)	3.18
LOC_Os02g27760	RPS15a	UP	IE (103.02)	724.23	UP	IE (6.01)	6.01
LOC_Os03g01900	RPS17	UP	IE (145.12)	252.99	UP	IE (18.42)	18.42
LOC_Os03g58050	RPS18	UP	IE (819)	885.39	UP	IE (3.49)	9.76
LOC_Os07g07719	RPS18a	UP	IE (266.91)	1068.83	UP	IE (3.62)	3.91
LOC_Os07g07770	RPS18b	UP	IE (318.11)	318.11	UP	IE (4.55)	84.05
LOC_Os03g31090	RPS19	UP	IE (166.16)	332.04	DOWN	–	–
LOC_Os03g46490	RPS21	UP	IE (53.78)	96.45	UP	IE (13.65)	13.65
LOC_Os03g60400	RPS23	UP	IE (3.37)	3.37	UP	IE (23.68)	164.02
LOC_Os10g20910	RPS23a	UP	IE (58.99)	439.57	UP	IE (5.46)	5.46
LOC_Os01g52490	RPS24	UP	IE (238.23)	238.23	UP	IE (32.20)	32.20
LOC_Os08g44480	RPS25	UP	IE (129.19)	129.19	UP	IE (14.58)	14.58
LOC_Os11g05562	RPS25a	UP	IE (91.95)	91.95	UP	IE (34.99)	34.99
LOC_Os01g60790	RPS26	UP	IE (143.50)	143.50	UP	IE (3.25)	6.94
LOC_Os04g27860	RPS27	UP	IE (552.55)	552.55	UP	IE (22.45)	22.45
LOC_Os01g22490	RPS27a	UP	IE (216.54)	1420.60	UP	L (9.35)	9.35
LOC_Os10g27174	RPS28	UP	IE (26.19)	543.29	UP	IE (33.77)	33.77
LOC_Os03g56241	RPS29	UP	IE (24.27)	47.44	UP	IE (42.79)	50.48
LOC_Os02g56014	RPS30	UP	IE (65.93)	65.93	UP	IE (13.00)	15.40
**H**_2_**0**_2_							
LOC_Os02g18550	RPS3a	UP	IE (8.8)	83.86	UP	IE (3.46)	3.46
LOC_Os01g25610	RPS4	UP	IE (29.68)	29.68	DOWN	–	–
LOC_Os05g30530	RPS4a	UP	IE (18.75)	487.002	UP	IE (9.86)	10.73
LOC_Os01g01060	RPS5	DOWN	–	–	UP	IE (22.29)	22.29
LOC_Os11g29190	RPS5a	UP	IE (33.87)	33.87	UP	E (6.49)	14.245
LOC_Os01g12090	RPS6	UP	IE (6.60)	53.10	UP	IE (8.03)	14.35
LOC_Os03g27260	RPS6a	UP	IE (12.95)	69.88	DOWN	–	–
LOC_Os02g21900	RPS7	UP	IE (16.64)	180.48	UP	L (3.00)	3.00
LOC_Os03g18570	RPS7a	UP	IE (10.30)	15.94	UP	IE (7.42)	9.804
LOC_Os07g43510	RPS9	UP	IE (4.76)	269.65	DOWN	–	–
LOC_Os03g05980	RPS9-2	UP	IE (33.16)	329.89	UP	IE (5.18)	5.18
LOC_Os01g73160	RPS10	UP	IE (10.29)	93.11	UP	L (5.65)	5.65
LOC_Os02g34460	RPS10a	UP	IE (16.59)	87.74	UP	IE (11.69)	11.69
LOC_Os07g38540	RPS13	UP	IE (11.59)	69.70	DOWN	–	–
LOC_Os08g02400	RPS13a	UP	IE (31.08)	137.24	DOWN	–	–
LOC_Os07g08660	RPS15	UP	IE (5.98)	51.09	UP	E (3.31)	6.49
LOC_Os02g27760	RPS15a	UP	IE (23.67)	298.48	UP	L (94.29)	4.29
LOC_Os03g01900	RPS17	UP	IE (4.18)	154.85	UP	L (7.78)	7.78
LOC_Os03g58050	RPS18	UP	IE (15.43)	187.73	UP	L (6.04)	6.04
LOC_Os07g07719	RPS18a	UP	IE (4.07)	491.19	DOWN	–	–
LOC_Os07g07770	RPS18b	UP	IE (6.82)	62.92	UP	L (4.56)	4.56
LOC_Os03g31090	RPS19	UP	IE (18.42)	71.76	DOWN	–	–
LOC_Os03g46490	RPS21	UP	IE (3.07)	81.90	UP	E (8.869)	11.50
LOC_Os03g60400	RPS23	UP	L (3.08)	3.08	UP	E (3.14)	14.99
LOC_Os10g20910	RPS23a	UP	IE (4.10)	164.71	DOWN	–	–
LOC_Os01g52490	RPS24	UP	IE (5.27)	66.26	UP	IE (10.69)	23.09
LOC_Os08g44480	RPS25	UP	IE (30.38)	58.89	UP	E (5.60)	7.79
LOC_Os11g05562	RPS25a	UP	IE (146.44)	155.55	UP	IE (4.65)	34.66
LOC_Os01g60790	RPS26	UP	IE (4.00)	47.17	DOWN	–	–
LOC_Os04g27860	RPS27	UP	IE (27.00)	66.18	UP	L (7.89)	23.34
LOC_Os01g22490	RPS27a	UP	IE (3.54)	146.72	DOWN	–	–
LOC_Os10g27174	RPS28	UP	IE (19.92)	137.66	UP	IE (78.78)	78.78
LOC_Os03g56241	RPS29	UP	IE (22.81)	37.30	UP	E (4.293)	16.65
LOC_Os02g56014	RPS30	UP	IE (3.12)	30.58	UP	IE (9.01)	9.01

*The regulation of all the 34 RPS genes in response to ABA, PEG, NaCl, and H_2_O_2_ in shoot and root are listed. Whether they are up-regulated or down-regulated, is mentioned separately. The genes having fold change ≥ 3 are considered to be up-regulated and the rest are considered to be down-regulated. Their extent of response is indicated as well with the fold change at that point. IE- immediate early genes (showing response within 15 min-3 h); E- early genes (showing response within 3 h- 12 h) and L- late responsive genes (showing response after 12 h post treatment). The overall maximum fold change for a gene under a specific treatment is also mentioned*.

### Differential expression of RPS genes upon infection with *Xanthomonas oryzae* pv. o*ryzae* (*Xoo*) and *Rhizoctonia solani*

Apart from the four abiotic stresses, the expression level of 34 RPS genes was also studied in response to the treatments with *Xoo* and *R. solani* pathogens that cause very serious Bacterial Leaf Blight (BLB) and Sheath Blight (SB) diseases, respectively in rice. In the BLB infected samples, 14 (40%) genes were down-regulated, and the rest of them were up-regulated (60%). *RPS6a* (26 fold), *RPS9* (18 fold), *RPS10a* (29 fold), and *RPS4* (13 fold) showed a high level of transcript upregulation when compared with the other up-regulated genes.

**Figure 6:**
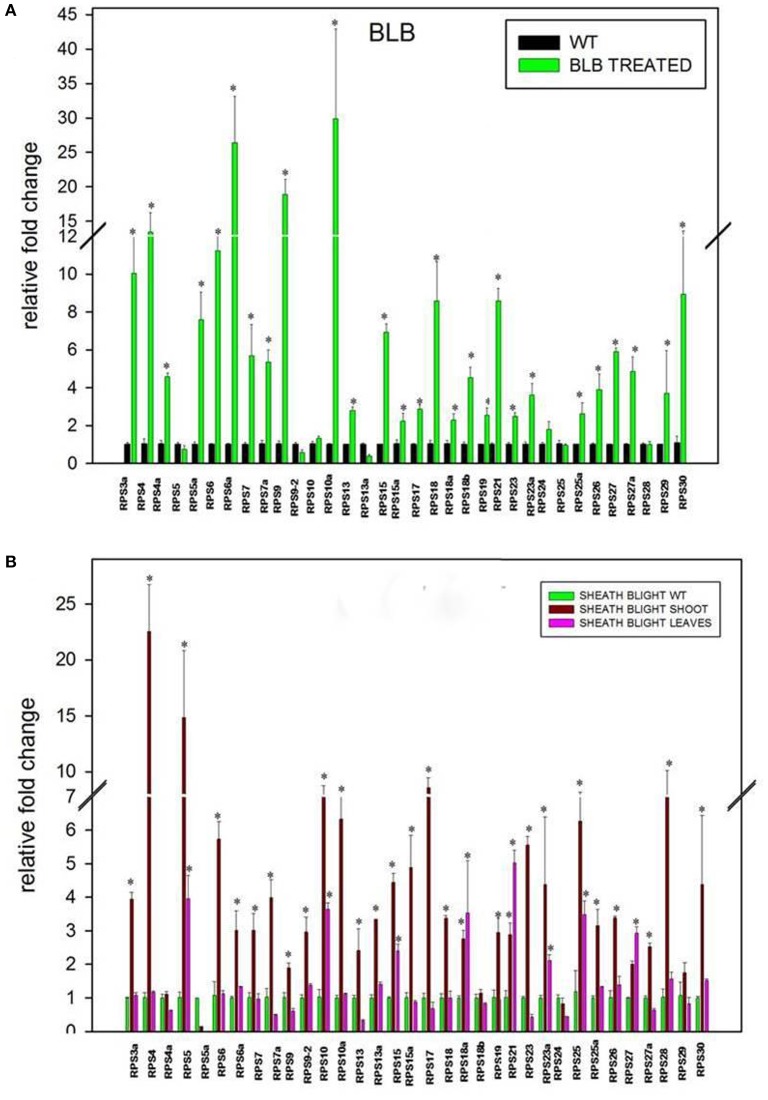
Differential expression of RPS genes in response to *Xanthomonas oryzae* pv. *oryzae* (*Xoo*) **(A)** and *Rhizoctonia solani* **(B)**, which cause Bacterial Leaf Blight (BLB) and Sheath Blight (SB) respectively. The expression of 34 RPS genes was determined in BLB and SB infected leaf samples after 20 and 25 d respectively after infection. The expression was normalized with corresponding untreated samples in the identical conditions. The statistical significance of expression analysis has been represented with asterisks ^*^P < 0.05.

The qRT-PCR analysis of 34 RPS genes was performed from both the shoot and the leaf samples infected with *R. solani*. The up-regulation of genes was observed particularly in the shoot tissues for a majority of the RPS genes. Thirteen (37%) genes were down-regulated in response to this pathogen in the shoot region whereas the 22 RPS (63%) genes were up-regulated. Among the genes that were expressive, *RPS4* and *RPS5* have exhibited 22 and 14 fold up-regulation, respectively, which is the highest compared with the other genes. The expression pattern of all the genes under two biotic stresses has been depicted graphically in Figures 6A,B.

### Comparative expression analysis of RPL and RPS genes

**Figure 7:**
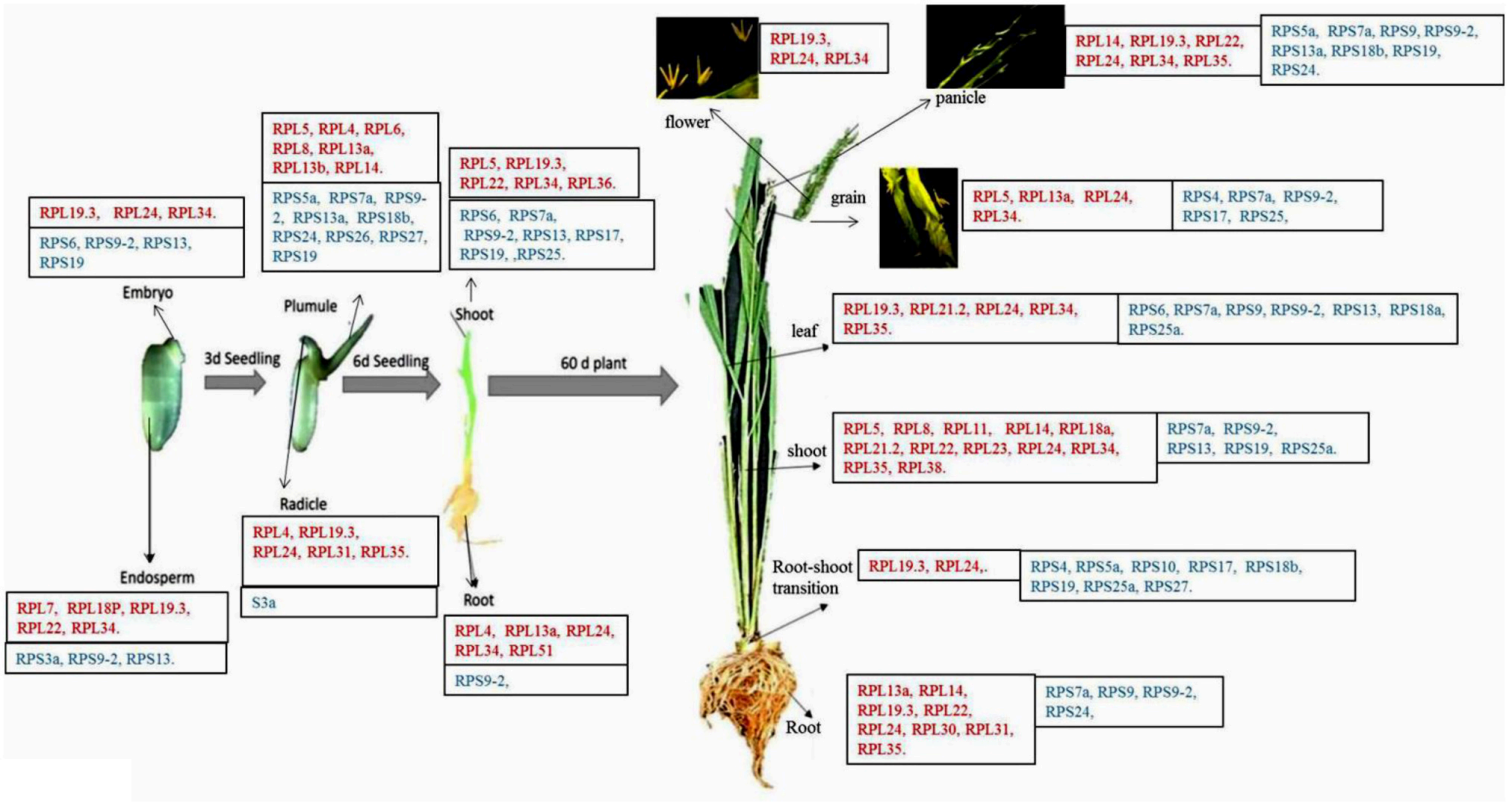
Native expression of RPL and RPS genes in different tissues. Some of the RPL and RPS genes showing considerably higher expression than the other genes in each of the 13 different tissues under native conditions have been listed in the boxes (Moin et al., 2016b). In each box, the names of the genes mentioned in red color belong to RPL gene family and the names of the genes mentioned in blue color belong to RPS gene family. The figure has been adapted and updated from Figure 3B of Moin et al. (2016b) under CC BY 4.0 license.

**Figure 8:**
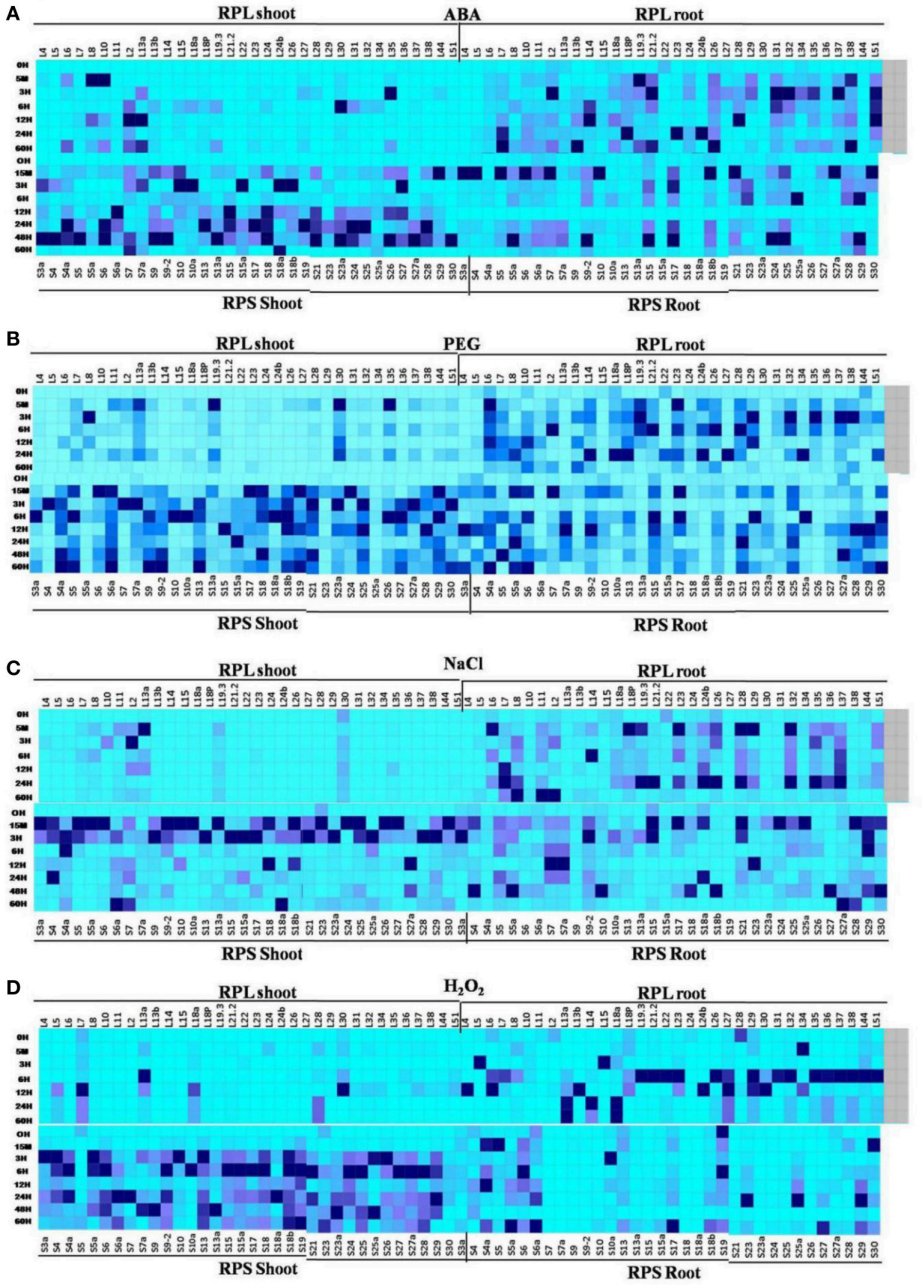
combined heat maps depicting differential expression of RPL and RPS genes under 4 abiotic stresses in shoot and root tissues. Differential expression of all the RPL and RPS genes under 4 abiotic stress is depicted together in 4 heat maps (**A**- ABA, **B**- PEG, **C**- NaCl, and **D**- H_2_O_2_). The mean of the fold change values of the biological and technical replicates are represented in the form of heat maps.

Previously available information about the RPL genes (Moin et al., 2016b) has been utilized and subsequently combined with the information obtained from the current study on the expression patterns of RPS genes. The details regarding their stress responsiveness and tissue-specific expression pattern under native condition have been summarized in tabular form to select suitable combinations of RPS and RPL genes for stress alleviation in transgenic plants. All the RPL and RPS genes up-regulated in response to four individual stress treatments were depicted in Supplementary Table 6. Highly up-regulated RPL and RPS genes in each of the 13 different tissues under native conditions have been represented in Figure 7. Four heat maps combining the expression of RPL and RPS genes under four abiotic stresses have been represented separately (Figures 8 A–D) and Supplementary Figure 6 shows the comparative expression analysis of both RPL and RPS genes under 13 different tissues in the form of a heat map.

One of the intriguing observations from this comparative analysis is that majority of the RPL genes were up-regulated in both shoots and roots of the plant under native conditions. But under all abiotic stress conditions, the maximum up-regulation was observed primarily in roots than in shoots. On the contrary, under the native conditions, the expression of RPS genes was mostly confined to shoots and the leaves compared to roots. Taken together, the up-regulation of the RPS genes and RPL genes was predominantly noticed in shoots and roots, respectively at all the time intervals under all the stress conditions.

According to the previous studies, *RPL23A, L19.3*, and *L38* have shown to exhibit a high level of expression under ABA, PEG and NaCl treatments. *RPL6, 12, 23A, 18A*, and *13A* have maintained high up-regulation under H_2_O_2_ treatment (Moin et al., 2016b). In the present study, *RPS13a, 18a, 4*, and *4a* have shown significant up-regulation in almost all the stresses in shoots, while *RPS29* and *RPS28* were highly expressive in roots. In the case of RPL genes, NaCl caused down-regulation of a majority of genes when compared to ABA and PEG. On the contrary, almost all the RPS genes showed up-regulation under salt stress in shoots. The H_2_O_2_ treatment caused down-regulation of 60 and 80% of RPL genes in shoots and roots, respectively when compared with MeJA, SA and cold treatments, which induced up-regulation of many of them. Most of the RPS genes were up-regulated under all the stresses in shoots, whereas in roots, 8, 22, 5, and 28% of the RPS genes were down-regulated under ABA, PEG, NaCl, and H_2_O_2_ treatments, respectively. The H_2_O_2_ treatment induced the down-regulation of many of them. *RPL30, 44, 22, 14, 29, 13A*, and *15* were down-regulated under ABA, PEG, and NaCl treatments and *RPS27a, 10, 20, 6*, and *9* were down-regulated in response to all the treatments (ABA, PEG, NaCl, and H_2_O_2_). *RPL10* was significantly activated upon infection with *Xoo* pathogen, whereas among the RPS genes, *RPS4* became commonly up-regulated under both *Xoo* and *R. solani* treatments.

## Discussion

Abiotic and nutrient stresses affect global agriculture by drastically reducing crop yield and productivity. Keeping the expanding world population in mind, stress management is one of the growing concerns for researchers worldwide. Rice is one of the most important staple food crops feeding a large portion of the world's population and is highly affected by some abiotic stresses, which include salinity, extreme temperatures, drought (Gao et al., 2007) and assailants like pests, fungi, virus and bacteria, etc. Being sessile, plants are highly vulnerable to these detrimental factors that drastically affect their growth and productivity. Hence, they have developed highly organized signaling responses and various other complex events to withstand and curtail the deleterious effects of these stress factors.

The role of RPs in ribosome assembly and house-keeping functions was very well recognized in several earlier studies on animal and plant systems. In addition to that, mutations in these RPs have led to physiological defects in several organisms. Effect on global protein synthesis due to these reduced RP functions could be one of the reasons behind developmental abnormalities. However, these physiological defects imply the potential extra-ribosomal and regulatory roles apart from the regular housekeeping functions for the RPs (Lu et al., 2015).

In this study, we have made comprehensive expression analyses of Ribosomal Protein Small subunit genes (RPS) in rice under multiple abiotic and biotic stresses and compared their expression with large subunit genes (RPL) genes (Moin et al., 2016b). Based on the differential and native expression data, we have short-listed the promising RPS and RPL combinations of candidate genes that might be useful in manipulating stress tolerance in rice. Also, we have presented an *in silico* analysis of the RPS protein properties and the putative promoter elements of the studied genes. With our previous study, which dealt with the extensive investigation on rice RPL genes (Moin et al., 2016b), we have tried to provide comprehensive information on the stress induced expression of all the ribosomal protein (RPS and RPL) genes in rice as a whole.

The Bacterial Leaf Blight (BLB) and Sheath Blight (SB) diseases are two of the major constraints in rice production with devastating influence on rice yield and its productivity (Zheng et al., 2013; Khan et al., 2014).

Availability of high-quality rice genome database helped us retrieve the genomic, CDS and protein sequences along with the 5′ upstream regions of the selected genes. A total of 56 RPS gene sequences were retrieved from the search in the databases, and finally, 34 genes were short listed excluding the identical members. The distribution of RPS genes appeared to be throughout the rice genome with each of the 12 chromosomes carrying at least one RPS gene family member with chromosome 3 exhibiting a maximum of 11 RPS genes. The RPs are mostly rich in positively charged amino acids, which can be correlated with the hypothesis that positively charged amino acids might play a significant role in electrostatic protein-RNA interactions (Law et al., 2006) within the ribosomal complex. Since the RPs are involved mostly in interactions that make up the ribosome complex along with rRNAs, the RPs are expected to have similar isoelectric pH range to facilitate their molecular interactions. Proteins with high isoelectric points tend to interact with DNA, RNA and other biomolecules that are negatively charged whereas the proteins with low isoelectric points prevent non-specific interaction with nucleic acids (Takakura et al., 2016). In a recent study, RPS6 has been reported to interact with a plant specific histone-deacetylase-2B and also with nucleosome assembly protein-1 thereby regulating the transcription of r-DNA in *Arabidopsis* (Son et al., 2015; Xiong and Sheen, 2015). Having a low isoelectric point might, therefore, be beneficial for RPS6 to minimize non-specific interactions. The RPs also have several domains associated with them like KOW, SPT2, HLH, CARD, ANTAR, HALZ, SANT domains etc. These domains have different functions including interaction with other proteins, transcription regulation, protein dimerization, apoptosis, binding to RNA and so on (Kyrpides et al., 1996; BouchierHayes and Martin, 2002; Shu and Zhulin, 2002; Jones, 2004; Novoseler et al., 2005; Feller et al., 2011).

Under native conditions in 13 different tissues, the RPS genes are prevalently up-regulated in 45 d old mature leaves, shoots and root-shoot transition and 7 d shoots and plumules and some of them were up-regulated in roots, panicles, embryo, endosperm and grains. But, none of them was found to be expressive in flowers. The role of RPS genes might be presumed to be involved in modulating developmental stages in vegetative tissues, mainly shoots and not to a greater extent in reproductive tissues. The activity of *RPS18A* promoter of *Arabidopsis* was seen in meristematic tissues and embryonic stage and mutations in these loci resulted in the formation of pointed leaves (Van Lijsebettens et al., 1994). This can be correlated with the transcriptional up-regulation of *RPS18, RPS18a*, and *RPS18b* in embryo, plumules, shoots, leaves, and root-shoot transition in the present study.

Around 60 and 20% of the genes were up-regulated immediately after treatment in shoots and roots, respectively under all the stress conditions. Up-regulation of these genes might fall under immediate response to the stress, and these genes are presumed to encode non-specific master regulators needed for plant core environmental stress response proteins. The immediate-early response to a certain stress might sometimes be non-specific, and responses with continuous up-regulation are considered to be specific ones (Kilian et al., 2007). And, the genes that are up-regulated at around 1 h time points can be assumed to have a pivotal role in early defense signaling and might have a function in reconstructing the proteins synthesis apparatus (Ouyang et al., 2007). This has led us to look for continuity in the up-regulation of immediate-early responsive RPS genes.

There are a considerable number of genes for which the up-regulation is maintained throughout the duration of the stress for example, *RPS13a, 18a, 4* and *4a*. Subsequently, *RPS29* has shown a remarkably high transcript level (≥100 fold) in roots under ABA and PEG treatments and also showed a moderate level of expression (11-fold) under H_2_O_2_ treatment. *RPS28* has consistently displayed a moderately high level of expression in all the stress conditions in root tissues.

On the other hand, *RPL23A, 6, 8, L19.3*, and *L38* among the members of large subunit genes have consistently shown up-regulation under ABA, PEG, NaCl treatments. *RPL18a, L24a, L24b, L30*, and *L34* were significantly overexpressed under oxidative stress. Moreover, *RPL23A, one* of the candidate genes that were identified using an activation tagging approach in water use efficiency (Moin et al., 2016a) might have a potential role to be utilized in modifying abiotic stress tolerance in rice orchestrating the responses of other candidate genes from both the subunits. Among the RPS genes, *RPS3a, RPS6, S15a, S17, S18a, S23a, S24, S25, S27a, and S28* are substantially up-regulated in shoots under a minimum of two abiotic stress conditions. *RPL10* and *RPS4* are significantly up-regulated under pathogen treatments. RPS18a has few significant protein domains associated with it and *RPS18a* has shown upregulation throughout all the stress treatments. Hence, the candidate genes overexpressed in small and large subunit categories listed in this study might be crucial in shortlisting genes to manipulate stress tolerance in rice by generating transgenic plants overexpressing the genes of both the subunits either individually or in combination.

It is crucial to look into the response of plants in the presence of multiple stress factors in order to completely evaluate the resistance responses of the candidate genes as the plant responses to a combination of stresses differ from individual stress responses (Atkinson and Urwin, 2012). In this context, the genes that are mostly up-regulated under all the stress conditions are significant. On the contrary, *RPS23* and *RPS7a* have maintained a low level of expression at all the time points under almost all the abiotic stresses in shoots, while *RPS19* has consistently exhibited down-regulation in roots under all the abiotic stress conditions. Except at certain time points under ABA treatment, *RPS27a, 10, 6*, and *9* have shown either down-regulation or no change in expression under rest of the stress conditions. *RPS18, 18a, 15*, and *13* have consistently displayed a low level of expression irrespective of the abiotic stress condition.

Apart from the genes that have been up-regulated in response to stress, there are also certain genes (mentioned above) that did not respond to the stress factors effectively in the respective tissues, probably indicating the limitations of their roles in stress tolerance. Under biotic stress, *RPS4, 10a*, and *6a* were up-regulated in BLB infected leaf samples, and *RPS4* and *5* were up-regulated in sheath blight infected leaf samples. Since *RPS4* has been up-regulated remarkably under all the abiotic and biotic stress conditions; it can be considered as one of the important RPS genes having both abiotic and biotic stress responsive roles.

The c*is*-regulatory elements (CREs) in the 5' UTR regions play significant roles in activation and suppression of gene expression that can confer tolerance to stresses (Ibraheem et al., 2010; Hernandez-Garcia and Finer, 2014). Throughout the putative promoter regions of almost all the RPS genes, regulatory elements that respond to abiotic and biotic stress treatments are distributed in multiple copies justifying their enhanced expression under various stress treatments.

The alarming change in climatic conditions will have a drastic consequence on global agriculture providing impetus to the already existing abiotic and biotic stresses. Temperature increase is more likely to be accompanied by recurrent flood, drought, enhanced salinity and prevalent occurrence of pathogen attacks (Atkinson and Urwin, 2012). This propels the researchers to produce transgenic plants with better yielding traits, which can withstand the altered environmental conditions. Ribosomal protein genes are important in this context because ribosome constitutes the protein synthesis machinery in a cell and under unfavorable conditions, the composition of the ribosome has been thought to change in order to assist biased protein translation (Wang J. et al., 2013). Moreover, some of the Genome-wide RNA sequencing technology and microarray analyses of other crop plants like Sorghum and alfalafa also revealed ribosomal protein genes to be important in response to abiotic stress (Fracasso et al., 2016; Liu et al., 2017).

Peptide mass spectrometry provides information that RPs of both prokaryotes and eukaryotes undergo a high degree of post-translational modifications (Arragain et al., 2010). Six RPs of *Escherichia coli* are methylated, three are acetylated, and one of them is methyl-thiolated. Although these modifications might have specific regulatory functions, the actual significance of these modifications is unclear (Nesterchuk et al., 2011). In plants, RPs are mostly methylated and methylation appears to be important for ribosome biogenesis and perhaps, its function (Friso and van Wijk, 2015). Apart from methylation, phosphorylation and glycosylation also play critical roles in post-translational modification of ribosomal proteins. Modulation of diurnal protein synthesis in plants has been predicted to have some link with differential phosphorylation of some ribosomal proteins. In higher plants, phosphorylation status of ribosomal protein S6 contributes to rapid adjustments of growth under environmental changes. Auxin enhances S6 protein phosphorylation in maize, which selectively increases ribosomal protein synthesis (Turkina et al., 2011). In terms of glycosylation, N glycosylation of Asn165 residue of Rps3, which is secreted in cancer cell lines, regulates the migration and invasive phenotype of the cancer cells (Kim et al., 2016). Overall the post-translational modification of RPs is important for the accuracy of the decoding machinery (Arragain et al., 2010) and it might also establish an additional RNA-protein contact which might be beneficial (Polikanov et al., 2015).

Differential expression of RPL and RPS genes under abiotic and biotic stresses, as we have described in the present study, indicates their role in stress responsiveness and might also modulate stress tolerance. The RPL and RPS genes that are remarkably up-regulated in response to almost all the stresses in comparison to the other genes can be considered as the cardinal RP genes that are essential to maintain the integrity of ribosomal units as well as ensuring sustained and undisturbed protein synthesis even under stress. This study provides an insight into the changes in the expression of RP genes in response to the environmental condition. Thus, the stress responsive increase in expression of several ribosomal protein genes can be considered for further exploitation as a resource in crop improvement programs.

## Author contributions

AS and SD performed the experiments. AS, MM, and PK designed the experiments and prepared the manuscript. AB and MD helped in qRT-PCR experiments and analysis. MSM performed the *Xanthomonas oryzae* pv. *oryzae* and *Rhizoctonia solani* infection on rice plants. PK supervised the work. AS, MM, and PK edited the manuscript.

### Conflict of interest statement

The authors declare that the research was conducted in the absence of any commercial or financial relationships that could be construed as a potential conflict of interest.

## Footnotes

^1^http://rice.plantbiology.msu.edu/index.shtml

^2^https://phytozome.jgi.doe.gov/pz/portal.html

^3^https://blast.ncbi.nlm.nih.gov/Blast.cgi

^4^https://www.ncbi.nlm.nih.gov/Structure/cdd/wrpsb.cgi

^5^http://pfam.xfam.org/

^6^http://orygenesdb.cirad.fr/tools.html

^7^http://plants.ensembl.org/Oryza_indica/Tools/Blast?db=core;tl=dxXz9p4PlES2NtPR-17828772

^8^https://www.arabidopsis.org/Blast/

^9^http://gsds.cbi.pku.edu.cn/

^10^http://bioinformatics.psb.ugent.be/webtools/plantcare/html/

^11^http://plantpan2.itps.ncku.edu.tw/promoter.php

^12^http://jaspar.genereg.net/cgi-bin/jaspar_db.pl?rm=browse&db=core&tax_group=plants

^13^https://psort.hgc.jp/form.html

^14^http://web.expasy.org/cgi-bin/protparam/protparam

^15^http://smart.embl-heidelberg.de/

^16^http://www.sbg.bio.ic.ac.uk/phyre2/html/page.cgi?id=index

^17^http://www.sbg.bio.ic.ac.uk/3dligandsite/

^18^http://www.genome.jp/tools/clustalw/

^19^http://www.megasoftware.net/

^20^https://software.broadinstitute.org/morpheus/

^21^http://bioinfo.ut.ee/primer3-0.4.0/

## Abbreviations

RPL: Ribosomal Protein Large subunit
RPS: Ribosomal Protein Small subunit
RP: Ribosomal proteins
ABA: Abscisic Acid
PEG: Polyethylene Glycol
NaCl: Sodium chloride
H_2_O_2_: Hydrogen peroxide
BLB: Bacterial Leaf Blight
SB: Sheath Blight.
