# Supplementary material for: Genome-Wide Identification and Comprehensive Expression Profiling of Ribosomal Protein Small Subunit (RPS) Genes and their Comparative Analysis with the Large Subunit (RPL) Genes in Rice
Source: Front Plant Sci. 2017 Sep 15;8:1553. doi: 10.3389/fpls.2017.01553 (PMC5605565; doi:10.3389/fpls.2017.01553)
Supplement: Supplementary file 1 [file DataSheet1.PDF]

Supplementary figure-1.

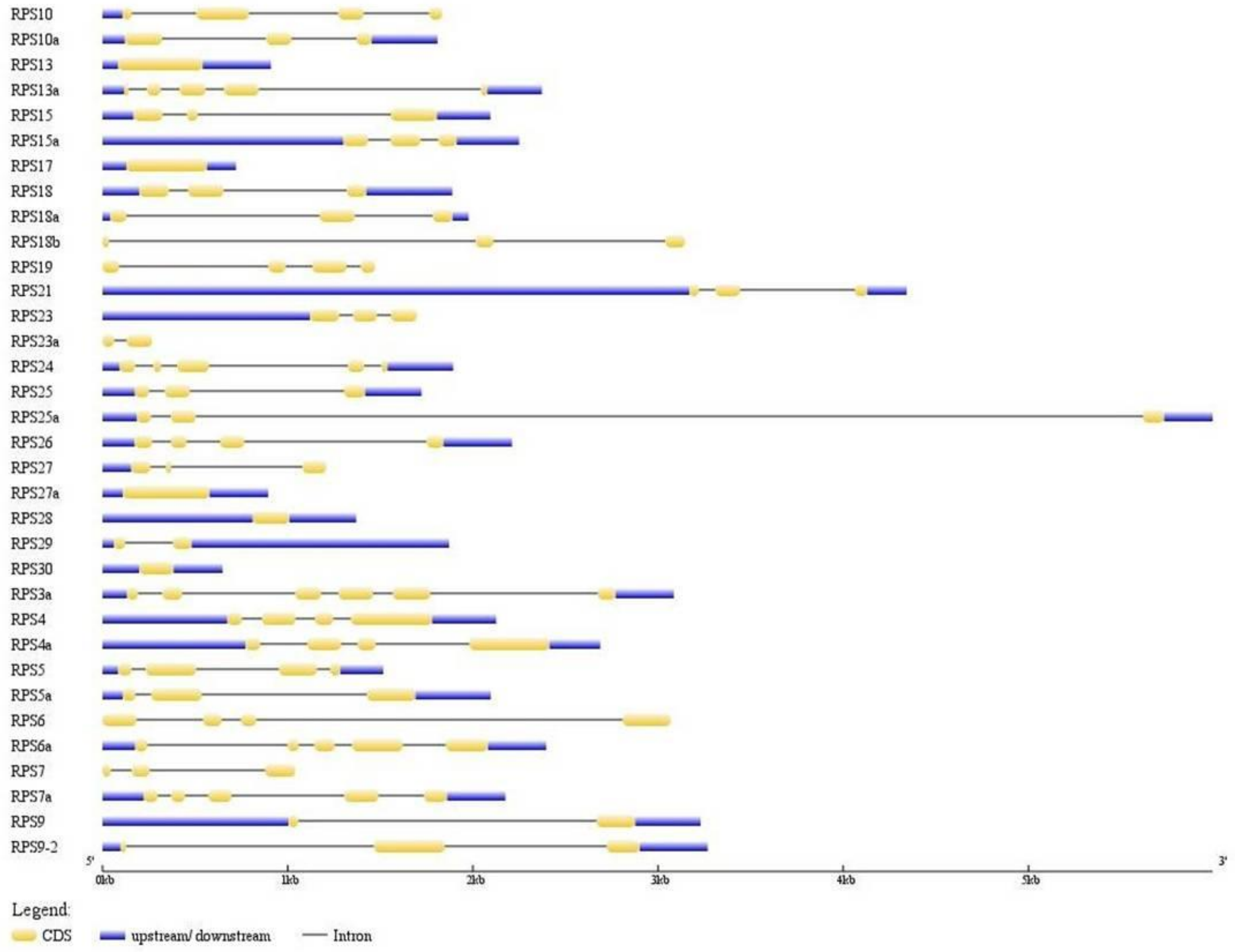

Gene structure of 34 RPS genes-

This diagram shows the inronic, exonic and UTRs of the 34 RPS genes. cDNA sequences of the selected 34 genes were submitted in the Gene Structure Display Server GSDSv2)[7]. Blue,yellow and line regions represent the upstream/downstream regions, exon and intron of the individual genes respectively.

Supplementary figure-2.

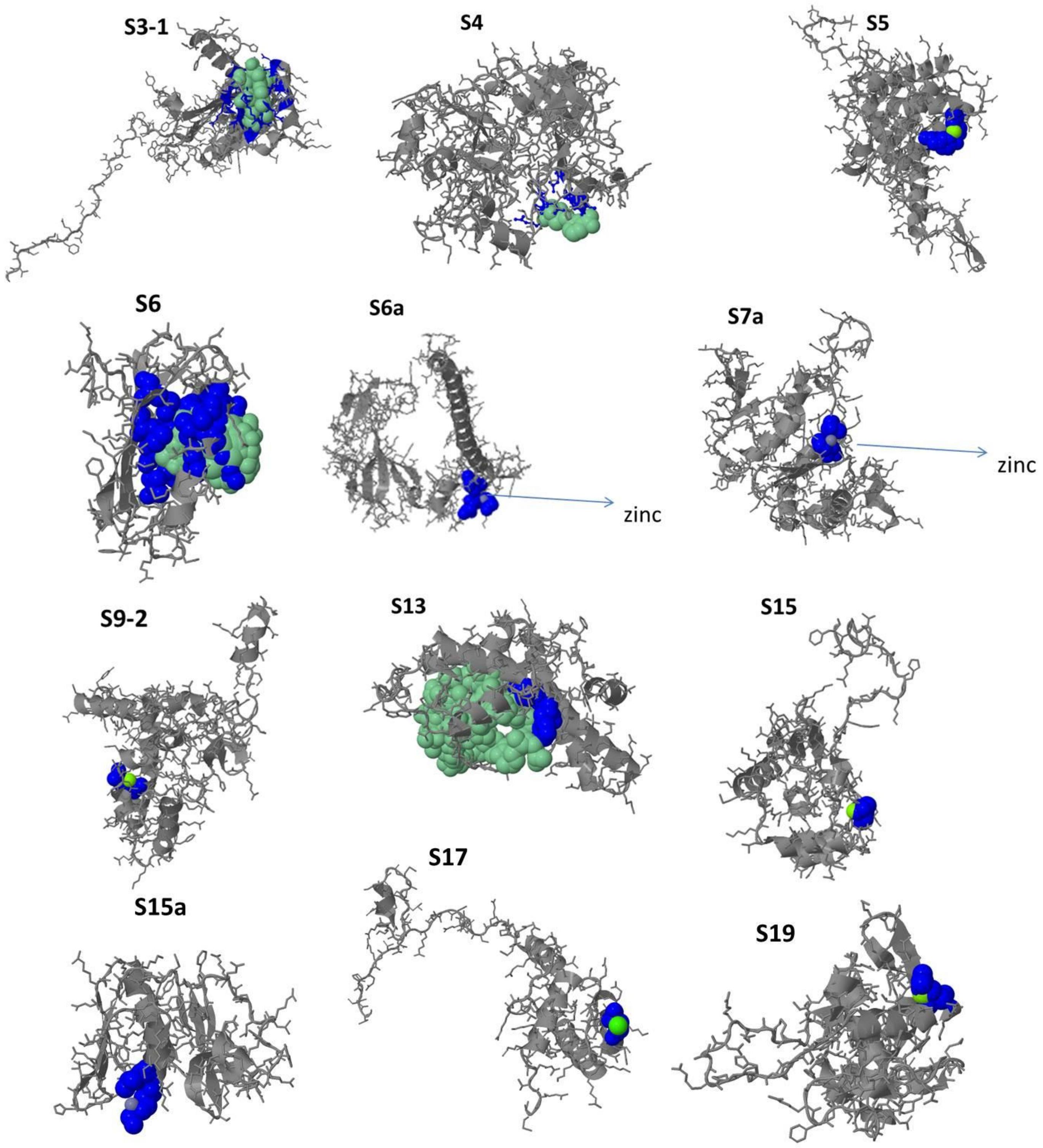

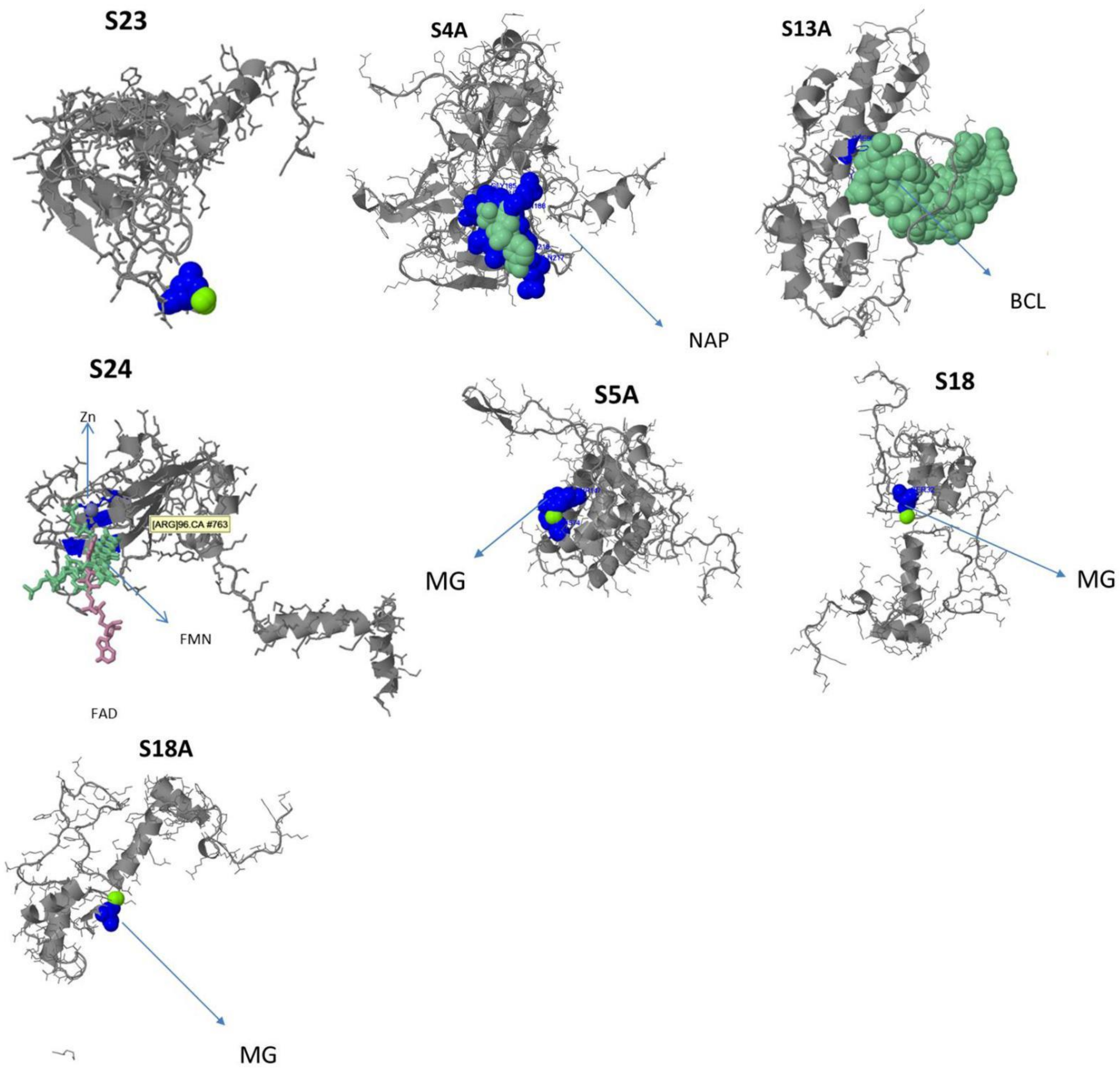

Predicted secondary structure and ligand binding sites of the RPS proteins-

To predict the three dimensional structure and to check for the presence of ligand binding sites on each RPS, we have used the Phyre2 and 3DLigandSite programs respectively. This figure shows the predicted secondary structure and ligand binding sites of the 19 RPS proteins with the metal ligands and cofactors mentioned in the images.

Supplementary Figure-3

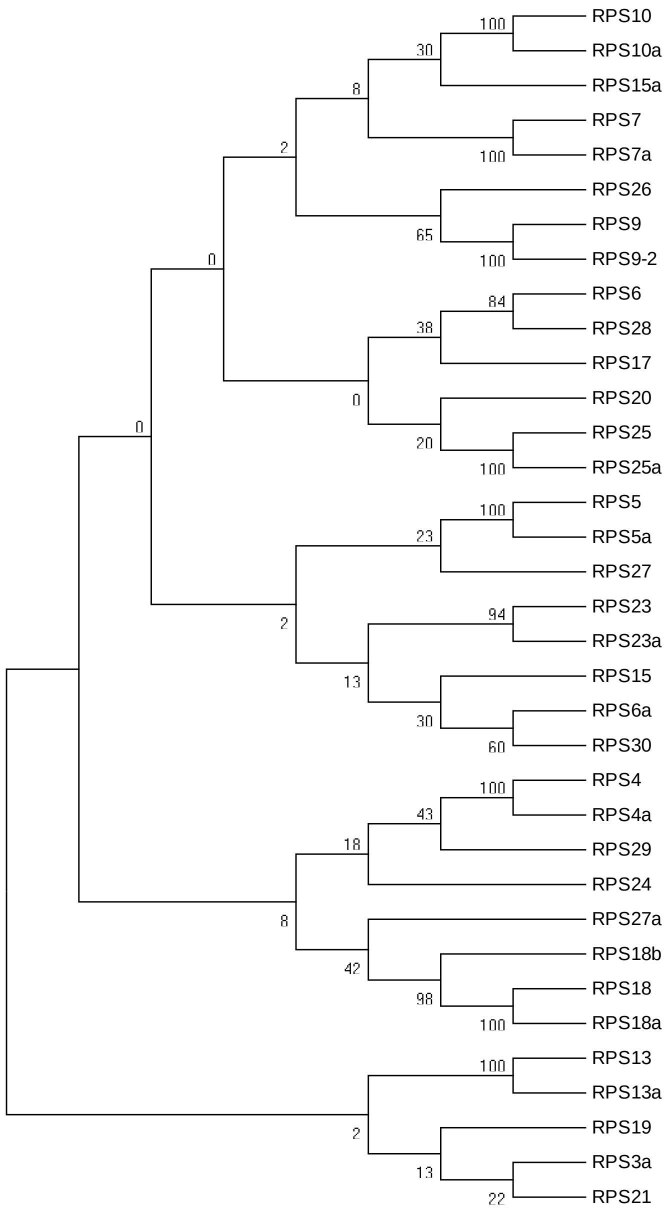

Phylogenetic tree showing relations within the RPS proteins.

Phylogenetic relationships among the 34 RPS proteins were deciphered by multiple sequence alignment in MEGA6[1] (Molecular Evolutionary Genetic Analysis) platform, which constructed an unrooted phylogenetic tree showing evolutionary sequence similarity among the proteins with bootstrap values of 100.

Supplementary figure- 3b

Among the divergent genes (RPS13a, 13, 3a, 19 and 21) from the above phylogenetic tree, RPS13 and RPS3a has 3 orthologous members which have been shown here in form of individual phylogenetic tree.

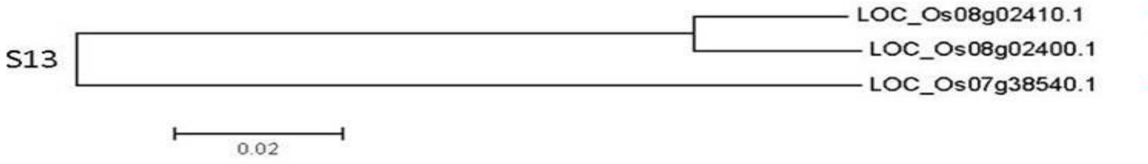

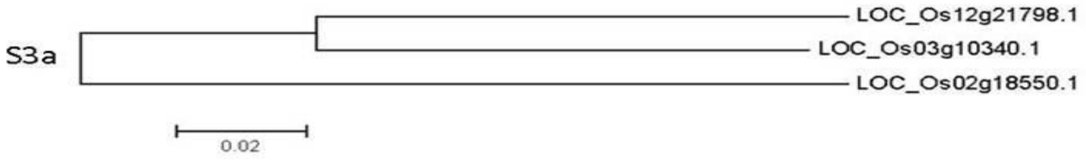

Supplementary Figure- 4.

a)

i) 15MIN SHOOT

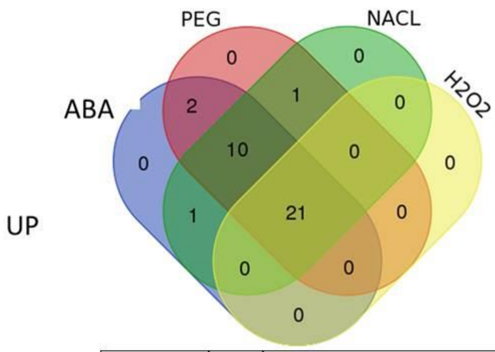

names	total	genes
ABA,H2O2, NACL PEG	21	S15a S30 S13 S20 S26 S23a S27a S9-2 S19 S3a S24 S4a S18b S9 S10 S6 S18 S18a S17 S15 S21
ABA NACL PEG	10	S29 S13a S10a S28 S25 S6a S5 S5a S4 S7
ABA PEG	2	S25a S7a
ABA NACL	1	S27
NACL PEG	1	S23

ii) 3H SHOOT

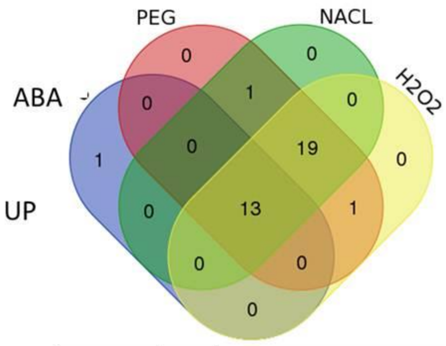

names	total	genes
ABA,H2O2, NACL PEG	13	S15a S13a S3a S25a S4a S27 S18b S10a S10 S7a S18a S4 S7
H2O2, NACL, PEG	19	S30 S13 S20 S29 S26 S23a S27a S9-2 S19 S28 S9 S25 S6a S5a S6 S18 S17 S15 S21
NACL, PEG	1	S5
H2O2, PEG	1	S24
ABA	1	S23

iii) 6H SHOOT

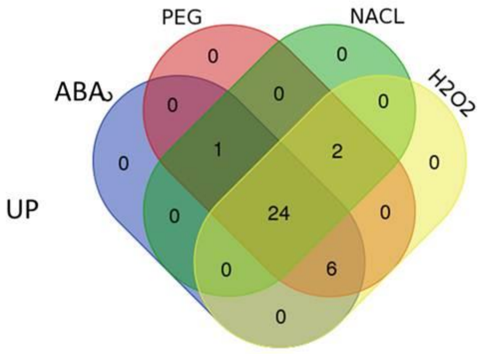

names	total	genes
ABA,H2O2, NACL PEG	24	S13 S20 S29 S13a S23a S27a S9-2 S19 S3a S25a S24 S4a S18b S28 S9 S25 S6a S5a S6 S18 S18a S17 S4 S15
ABA NACL PEG	1	S5
ABA H2O2 PEG	6	S26 S27 S10a S10 S7 S21
Nacl H2O2 PEG	2	S15a S30

iv) 12H SHOOT

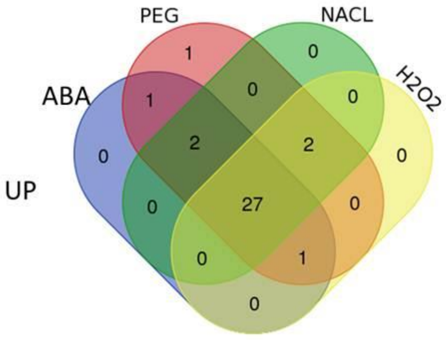

names	total	genes
ABA,H2O2, NACL PEG	27	S15a S20 S29 S26 S13a S23a S27a S19 S3a S24 S4a S27 S18b S28 S9 S10 S25 S6a S5a S6 S18 S18a S17 S4 S15 S7 S21
ABA NACL PEG	2	S25a S10a
ABA H2O2 PEG	1	S13
H2O2 NACL PEG	2	S30 S9-2
ABA PEG	1	S5
PEG	1	S7a

v) 24H SHOOT

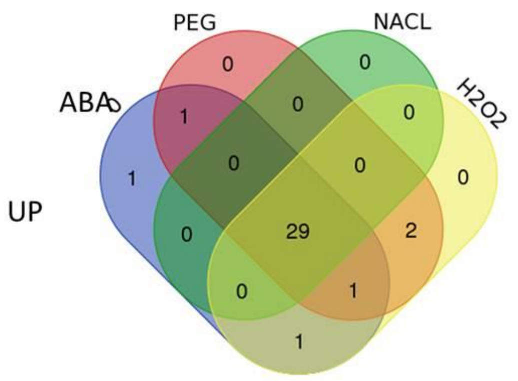

names	total	genes
ABA,H2O2, NACL PEG	29	S15a S13 S20 S29 S26 S13a S23a S27a S9-2 S19 S3a S25a S24 S4a S27 S18b S28 S9 S10 S25 S6a S5a S18 S18a S17 S4 S15 S7 S21
ABA H2O2 PEG	1	S6
ABA PEG	1	S5
ABA H2O2	1	S10a
H2O2 PEG	2	S30 S7a
ABA	1	S23

vi) 48H SHOOT

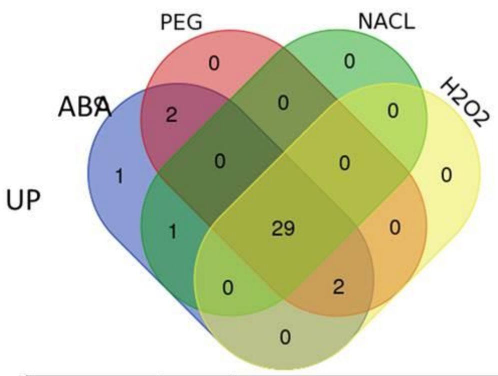

names	total	genes
ABA H2O2 NACL PEG	29	S15a S13 S20 S29 S26 S13a S23a S27a S9-2 S19 S3a S25a S24 S4a S27 S18b S28 S9 S10 S25 S6a S5a S6 S18 S18a S17 S4 S7 S21
ABA H2O2 PEG	2	S10a S15
ABA PEG	2	S30 S5
ABA NACL	1	S7a
ABA	1	S23

vii) 60H SHOOT

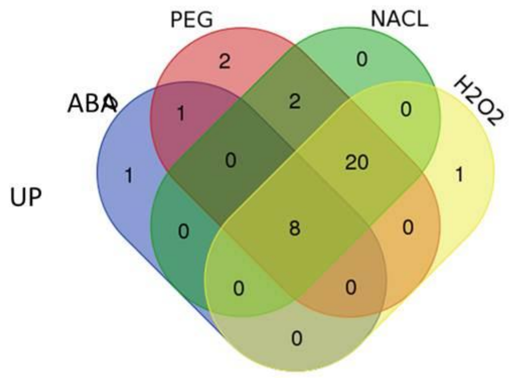

names	total	genes
ABA H2O2 NACL PEG	8	S15a S20 S13a S25a S10 S18a S4 S7
H2O2 NACL PEG	20	S13 S26 S23a S27a S9-2 S19 S3a S24 S4a S27 S18b S28 S9 S25 S6a S6 S18 S17 S15 S21
ABA PEG	1	S10a
NACL PEG	2	S30 S29
ABA	1	S7a
PEG	2	S5 S5a
H2O2	1	S23

b) i) 15 MIN ROOT

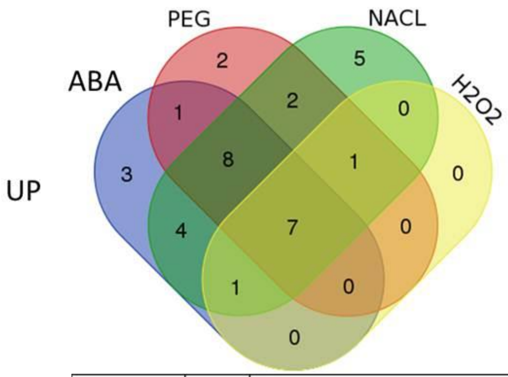

names	total	genes
ABA H2O2 NACL PEG	7	S30 S9-2 S3a S28 S7a S5 S6
ABA NACL PEG	8	S29 S23a S27 S25 S5a S17 S21 S7
ABA H2O2 NACL	1	S24
H2O2 NACL PEG	1	S4a
ABA PEG	1	S10
ABA NACL	4	S18b S9 S18 S4
NACL PEG	2	S23 S25a
ABA	3	S20 S27a S6a
PEG	2	S26 S10a
NACL	5	S15a S13 S13a S18a S15

ii) 3H ROOT

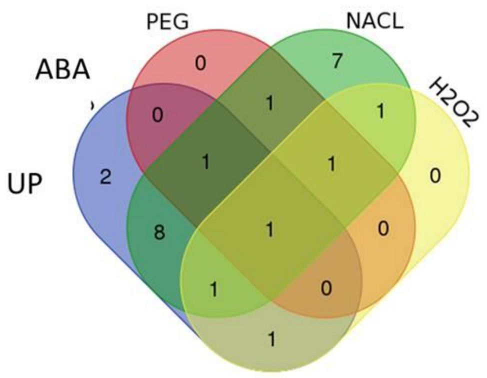

names	total	genes
ABA H2O2 NACL PEG	1	S28
ABA NACL PEG	1	S25
ABA H2O2 NACL	1	S25a
H2O2 NACL PEG	1	S7a
ABA NACL	8	S29 S23 S9-2 S17 S3a S4a S21 S27
ABA H2O2	1	S6
NACL PEG	1	S5
H2O2 NACL	1	S10a
ABA	2	S30 S23a
NACL	7	S13 S26 S13a S5a S24 S15 S7

iii) 6H ROOT

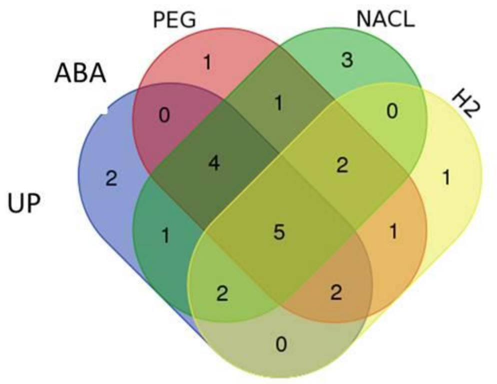

names	total	genes
ABA H2 NACL PEG	5	S28 S29 S23 S25a S4a
ABA NACL PEG	4	S10a S30 S13 S17
ABA H2 PEG	2	S6 S9-2
ABA H2 NACL	2	S25 S3a
H2 NACL PEG	2	S7a S5
ABA NACL	1	S5a
NACL PEG	1	S18b
H2 PEG	1	S15
ABA	2	S21 S27
PEG	1	S23a
NACL	3	S9 S26 S7
H2O2	1	S24

iv) 12H ROOT

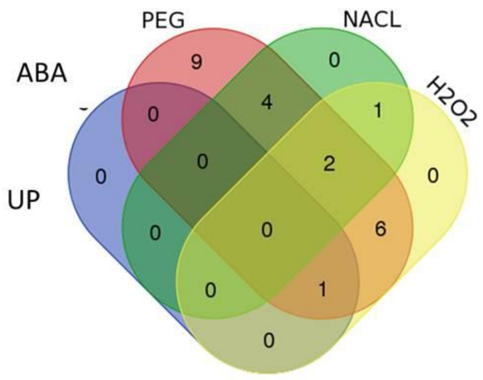

names	total	genes
ABA H2O2 PEG	1	S25a
H2O2 NACL PEG	2	S4a S21
NACL PEG	4	S28 S7a S23 S7
H2O2 PEG	6	S25 S29 S5a S6 S9-2 S15
H2O2 NACL	1	S24
PEG	9	S18b S15a S30 S13 S26 S23a S17 S3a S27

v) 24H ROOT

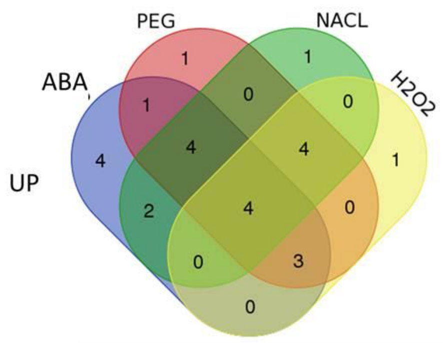

names	total	genes
ABA H2O2 NACL PEG	4	S5a S25a S21 S27
ABA NACL PEG	4	S15a S5 S7a S4a
ABA H2O2 PEG	3	S30 S25 S6
H2O2 NACL PEG	4	S28 S29 S23 S9-2
ABA PEG	1	S3a
ABA NACL	2	S13 S7
ABA	4	S18b S20 S26 S17
PEG	1	S23a
NACL	1	S18a
H2O2	1	S24

vi) 48H ROOT

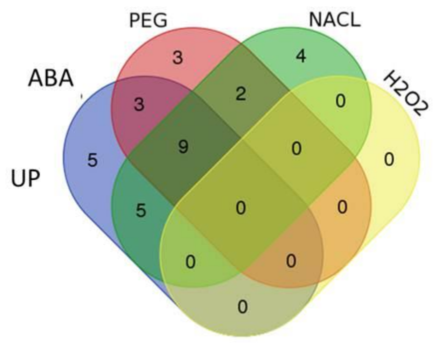

names	total	genes
ABA NACL PEG	9	S28 S25 S29 S5 S7a S17 S24 S7 S27
ABA PEG	3	S13 S6 S21
ABA NACL	5	S18b S30 S5a S9-2 S4a
NACL PEG	2	S18 S25a
ABA	5	S9 S20 S23a S3a S15
PEG	3	S15a S6a S27a
NACL	4	S10a S10 S23 S4

vii) 60H ROOT

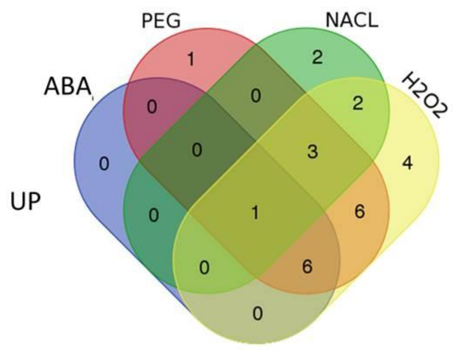

names	total	genes
ABA H2O2 NACL PEG	1	S28
ABA H2O2 PEG	6	S25 S29 S6 S25a S21 S27
H2O2 NACL PEG	3	S30 S5a S4a
H2O2 PEG	6	S15a S23 S9-2 S17 S3a S24
H2O2 NACL	2	S7a S7
PEG	1	S23a
NACL	2	S20 S27a
H2O2	4	S18b S10 S18 S15

Supplementary Figure-4 a and b-Venn diagrams showing expression overlap of up-regulated genes in shoot (a) and root (b) in response to all the four abiotic stresses in different time points. At a particular time points, the names of the genes upregulated under a combination of abiotic stresses are mentioned in the Venn diagrams and in the chart accompanied with them.

Supplementary Figure 5-

a)

i) 15MIN SHOOT

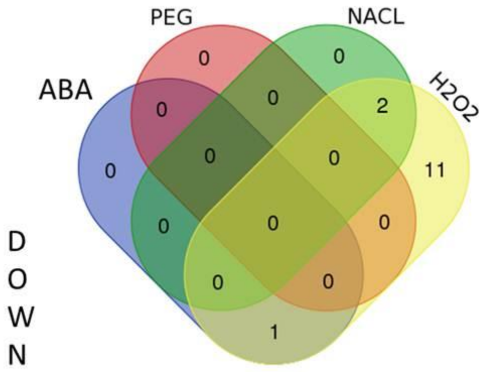

names	total	genes
ABA H2O2	1	S23
H2O2 NACL	2	S25a S7a
H2O2	11	S10a S28 S4 S25 S29 S6a S5 S13a S7 S27 S5a

ii) 3H SHOOT

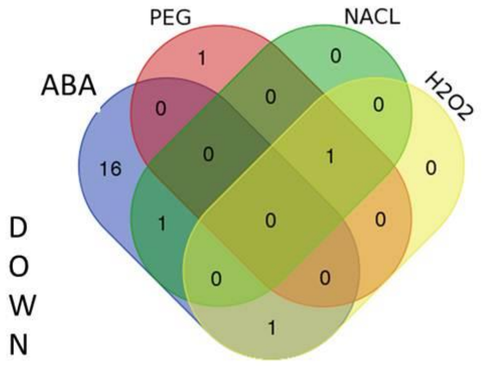

names	total	genes
H2O2 NACL PEG	1	S23
ABA NACL	1	S24
ABA H2O2	1	S5
ABA	16	S30 S28 S9 S25 S20 S29 S6a S26 S23a S5a S6 S27a S9-2 S19 S15 S21
PEG	1	S27

iii) 6H SHOOT

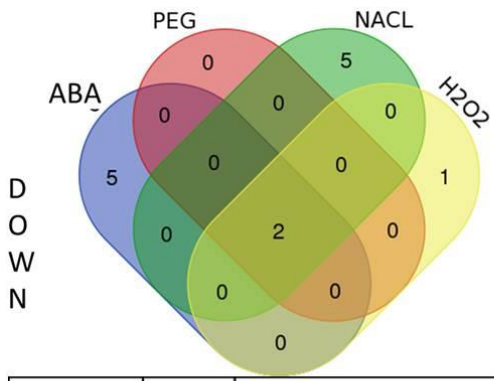

names	total	genes
ABA H2O2 NACL PEG	2	S23 S7a
ABA	5	S18 S15a S30 S13 S17
NACL	5	S10a S10 S26 S7 S27
H2O2	1	S5

iv) 12H SHOOT

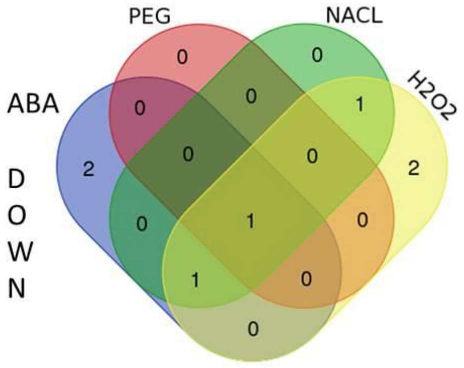

names	total	genes
ABA H2O2 NACL PEG	1	S23
ABA H2O2 NACL	1	S7a
H2O2 NACL	1	S5
ABA	2	S30 S9-2
H2O2	2	S25a S10a

v) 24H SHOOT

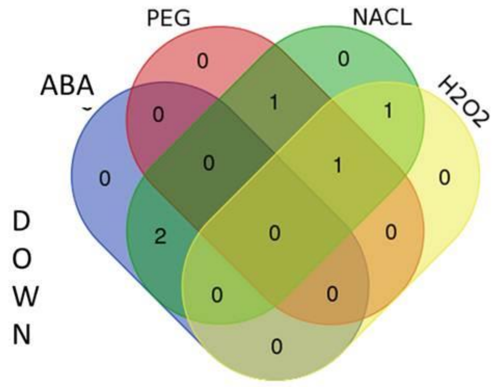

names	total	genes
H2O2 NACL PEG	1	S23
ABA NACL	2	S30 S7a
NACL PEG	1	S10a
H2O2 NACL	1	S5

vi) 48H SHOOT

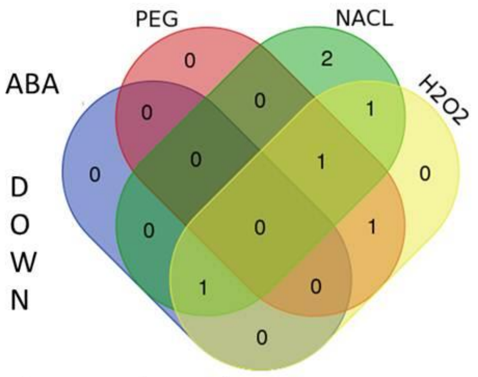

names	total	genes
ABA H2O2 NACL	1	S30
H2O2 NACL PEG	1	S23
H2O2 PEG	1	S7a
H2O2 NACL	1	S5
NACL	2	S15 S10a

vii) 60H SHOOT

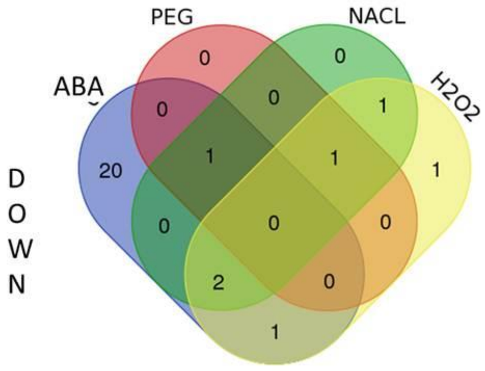

names	total	genes
ABA NACL PEG	1	S23
ABA H2O2 NACL	2	S5 S5a
H2O2 NACL PEG	1	S7a
ABA H2O2	1	S29
H2O2 NACL	1	S10a
ABA	20	S18b S13 S28 S9 S25 S6a S26 S23a S6 S18 S27a S9-2 S17 S19 S3a S24 S15 S4a S21 S27
H2O2	1	S30

b) i) 15 MIN ROOT

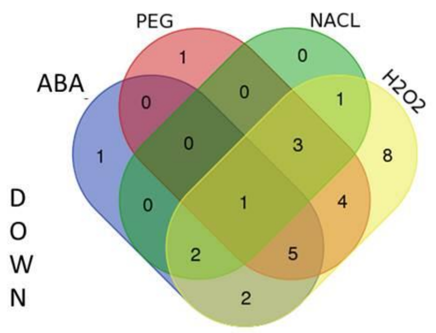

names	total	genes
ABA H2O2 NACL PEG	1	S19
ABA H2O2 PEG	5	S15a S13 S13a S18a S15
ABA H2O2 NACL	2	S10a S26
H2O2 NACL PEG	3	S20 S6a S27a
ABA H2O2	2	S23 S25a
H2O2 PEG	4	S18b S9 S18 S4
H2O2 NACL	1	S10
ABA	1	S4a
PEG	1	S24
H2O2	8	S25 S29 S23a S5a S17 S21 S7 S27

ii) 3H ROOT

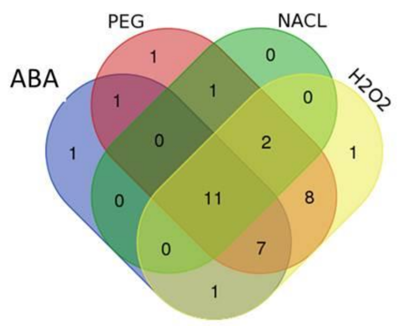

names	total	genes
ABA H2O2 NACL PEG	11	S15a S20 S27a S19 S18b S9 S10 S6a S18 S18a S4
ABA H2O2 PEG	7	S13 S26 S13a S24 S5a S15 S7
H2O2 NACL PEG	2	S30 S23a
ABA PEG	1	S10a
ABA H2O2	1	S5
NACL PEG	1	S6
H2O2 PEG	8	S29 S23 S9-2 S3a S4a S27 S17 S21
ABA	1	S7a
PEG	1	S25a
H2O2	1	S25

v) 6H ROOT

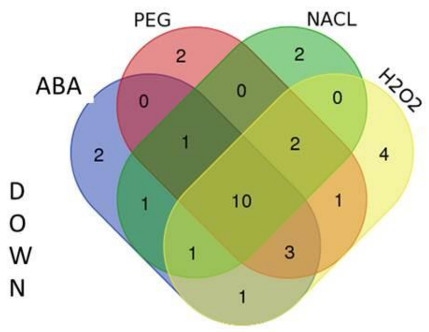

names	total	genes
ABA H2O2 NACL PEG	10	S15a S10 S20 S6a S13a S18 S18a S27a S4 S19
ABA NACL PEG	1	S24
ABA H2O2 PEG	3	S9 S26 S7
ABA H2O2 NACL	1	S23a
H2O2 NACL PEG	2	S21 S27
ABA NACL	1	S15
ABA H2O2	1	S18b
H2O2 PEG	1	S5a
ABA	2	S5 S7a
PEG	2	S25 S3a
NACL	2	S6 S9-2
H2O2	4	S10a S30 S13 S17

vi) 12H ROOT

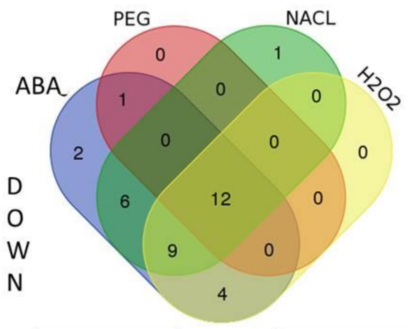

names	total	genes
ABA H2O2 NACL PEG	12	S20 S13a S27a S19 S10a S9 S10 S6a S5 S18 S18a S4
ABA H2O2 NACL	9	S15a S30 S13 S26 S23a S3a S27 S18b S17
ABA PEG	1	S24
ABA NACL	6	S29 S9-2 S25 S5a S6 S15
ABA H2O2	4	S23 S28 S7a S7
ABA	2	S4a S21
NACL	1	S25a

iii) 24H ROOT

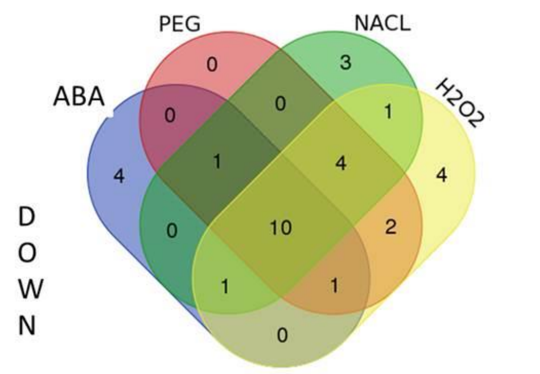

names	total	genes
ABA H2O2 NACL PEG	10	S10a S10 S9 S6a S13a S18 S27a S4 S19 S15
ABA NACL PEG	1	S24
ABA H2O2 PEG	1	S18a
ABA H2O2 NACL	1	S23a
H2O2 NACL PEG	4	S18b S20 S26 S17
H2O2 PEG	2	S13 S7
H2O2 NACL	1	S3a
ABA	4	S28 S29 S23 S9-2
NACL	3	S30 S25 S6
H2O2	4	S15a S7a S5 S4a

iv) 48H ROOT

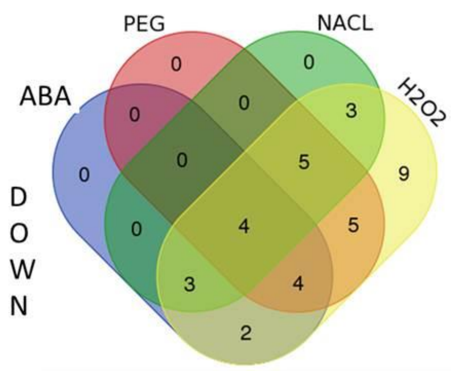

names	total	genes
ABA H2O2 NACL PEG	4	S26 S13a S18a S19
ABA H2O2 PEG	4	S10a S10 S23 S4
ABA H2O2 NACL	3	S15a S6a S27a
H2O2 NACL PEG	5	S9 S20 S23a S3a S15
ABA H2O2	2	S18 S25a
H2O2 PEG	5	S18b S30 S5a S9-2 S4a
H2O2 NACL	3	S13 S6 S21
H2O2	9	S28 S25 S29 S7a S5 S17 S24 S7 S27

vii) 60H ROOT

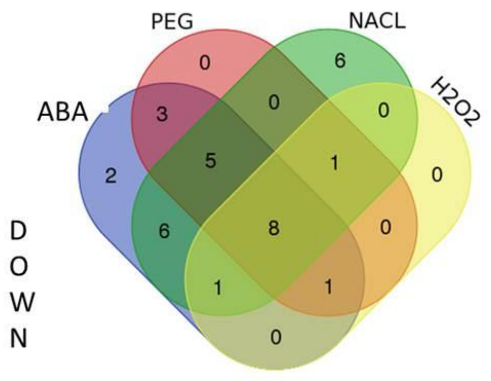

names	total	genes
ABA H2O2 NACL PEG	8	S10a S13 S9 S6a S26 S13a S18a S4
ABA NACL PEG	5	S18b S10 S18 S19 S15
ABA H2O2 PEG	1	S27a
ABA H2O2 NACL	1	S23a
H2O2 NACL PEG	1	S5
ABA PEG	3	S20 S7a S7
ABA NACL	6	S15a S23 S9-2 S17 S3a S24
ABA	2	S30 S4a
NACL	6	S25 S29 S6 S25a S21 S27

Supplementary figure 5a and 5b-

Venn diagrams showing expression overlap of down-regulated genes in shoot (a) and root (b) in response to all the four abiotic stresses in different time points. At a particular time points, the names of the genes down-regulated under a combination of abiotic stresses are mentioned in the Venn diagrams and in the chart accompanied with them.

Supplementary Figure- 6

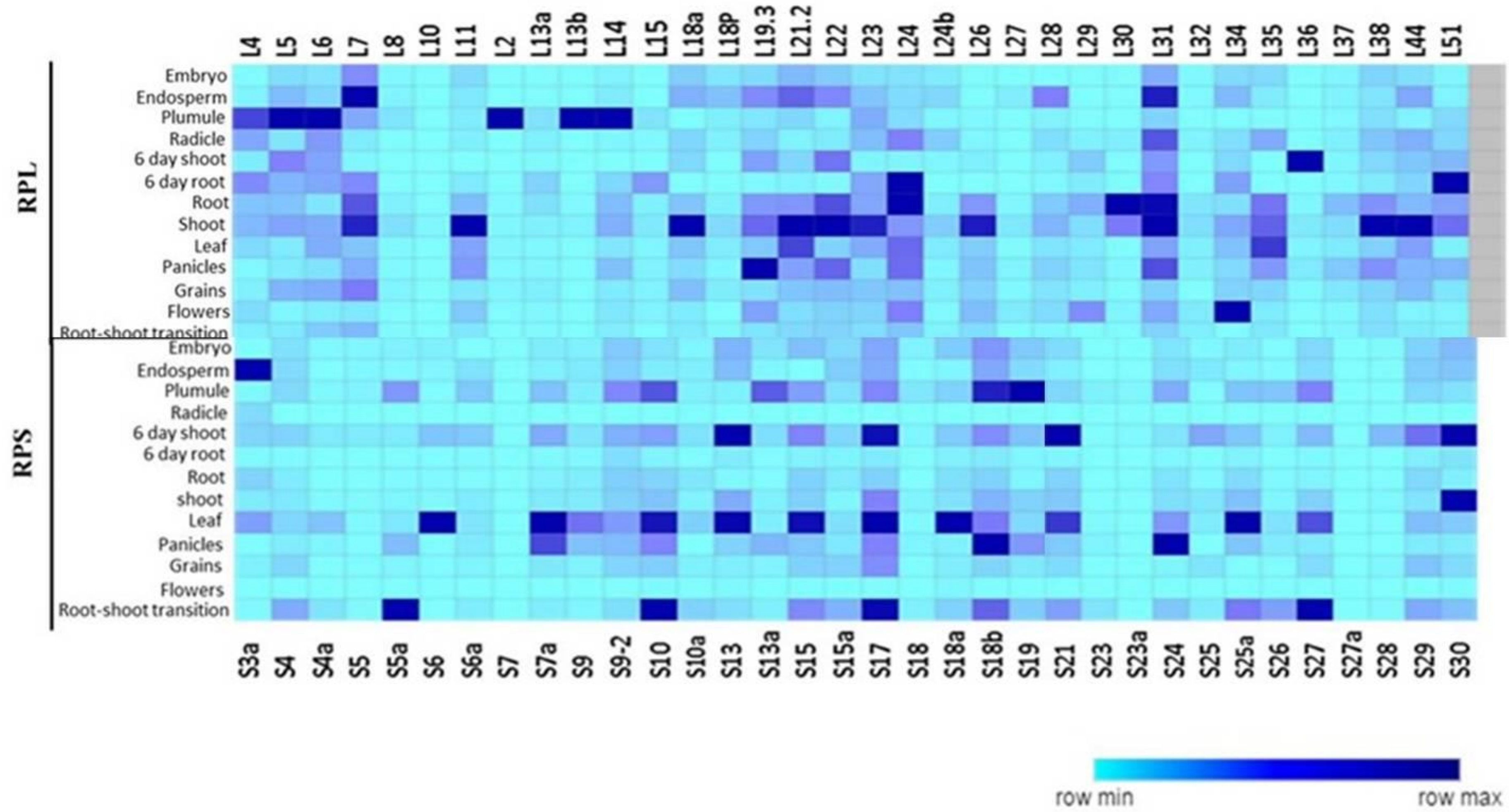

Supplementary figure-6- Combined heat map depicting the native expression of RPL and RPS genes in 13 different tissues

S

upplementary figure 7-

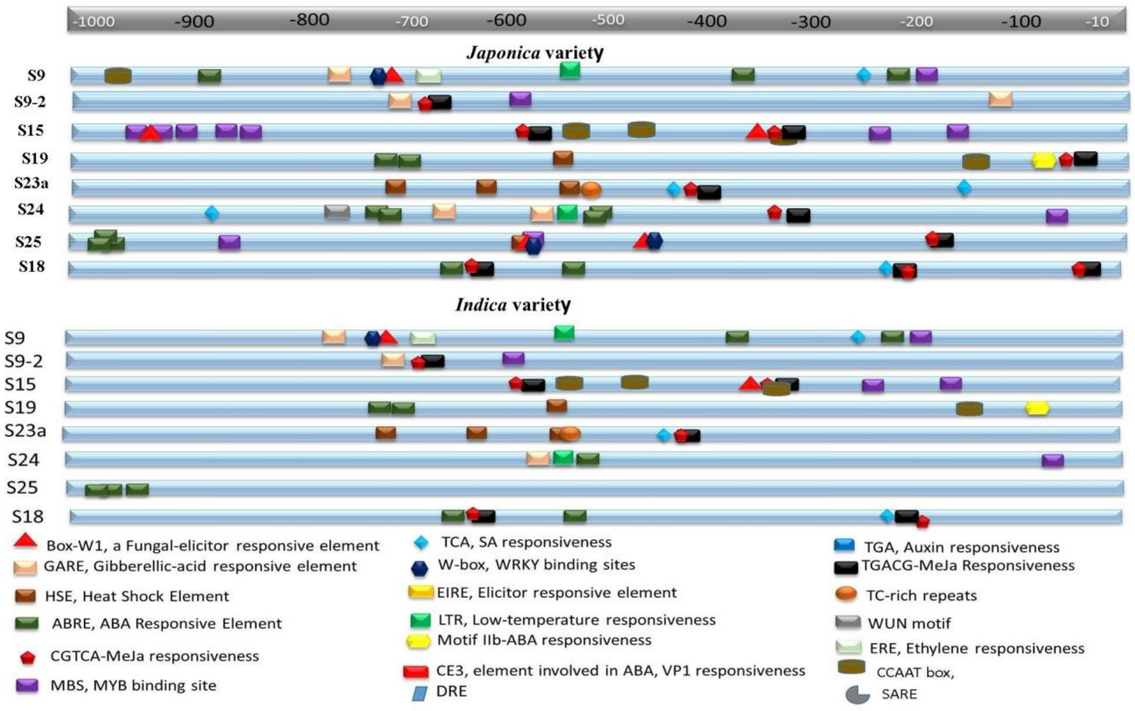

Supplementary figure 7- *Cis*- acting regulatory elements present on the promoter regions of the eight genes of *indica*and *japonica* varieties that showed less sequence similarity in their promoter regions.

Supplementary table 1- BLAST analysis of the coding and the 5'-upstream region of the 34 RPS genes in OrygeneDB and EnsemblPlants databases to check the similarity of the sequences in *indica*and *japonica* varieties.

**Gene**				**5' upstream region**		
Gene name	ODB (Japonica) Query coverage	ODB (Indica) Query coverage	Ensembl (Indica)	ODB (Japonica) Query coverage	ODB (Indica) Query coverage	Ensembl (Indica)
1. S3a LOC_Os02g18550.1	101.40	101.40	100%	99.9	99.9	98.2%
2. S4 LOC_Os01g25610.1	99.75	99.75	100%	98.5	98.5	99.5%
3. S5 LOC_Os01g01060.1	101.99	101.99	100%	100	100	100%
4. S6 LOC_Os01g12090.1	101.13	101.13	100%	99.90	99.90	100%
5. S6a LOC_Os03g27260.1	100.27	99.19	100%	99.90	99.90	98%
6. S7 LOC_Os02g21900.1	100.99	100.99	99.4%	99.90	99.90	98.9%
7. S7a LOC_Os03g18570.1	101.55	101.55	100%	99.90	99.90	99.1%
8. S9 LOC_Os07g43510.1	101.15	101.15	96.3%	99.90	99.90	98.5%
9. S9-2 LOC_Os03g05980.1	95.58	95.58	99.2%	99.90	99.90	98.6%
10. S10 LOC_Os01g73160.1	100.00	100.00	100%	99.90	99.90	100%
11. S13 LOC_Os07g38540.1	99.78	99.78	99.8%	99.9	99.9	99%
12. S15 LOC_Os07g08660.1	100	100	99.6%	99.9	99.8	98%
13. S15a LOC_Os02g27760.1	101.27	101.27	99.4%	99.90	99.90	99%
14. S17 LOC_Os03g01900.1	99.77	99.77	99.5%	99.90	99.90	98.8%
15. S19 LOC_Os03g31090.1	100.00	100.00	100%	67.00	47.00	98.5%
16. S21 LOC_Os03g46490.1	100.80	100.80	100%	99.90	99.90	100%
17. S23 LOC_Os03g60400.1	98.6	98.6	100%	62	63	98.65%
18. S23a LOC_Os10g20910.1	100	100	100%	99.88	106.7	97.5%
19. S24 LOC_Os01g52490.1	94	94	100%	99.9	99.9	99.4%
20. S25 LOC_Os08g44480.1	99.39	99.39	100%	76	69	100%
21. S26 LOC_Os01g60790.1	102.49	102.49	100%	99.90	99.90	99.2%
22. S27 LOC_Os04g27860.1	88.89	88.39	100%	99.90	99.90	98.9%

23. S27a LOC_Os01g22490.1	99.79	99.79	100%	99.89	99.89	100%
24. S28 LOC_Os10g27174.1	99.49	99.49	100%	99.9	99.9	99.7%
25. S29 LOC_Os03g56241.1	94.15	94.15	100%	99.92	99.92	99.3%
26. S30 LOC_Os02g56014.1	97.88	97.88	99.5%	99.89	99.89	99.3%
27. S10a LOC_Os02g34460.2	100.24	100.24	100%	99.9	99.9	98.77%
28. S4a LOC_Os05g30530.1	100.38	100.38	98.8%	99.8	98.30	98.8%
29. S13a LOC_Os08g02400.1	89.25	89.25	100%	99.50	99.40	100%
30. S25a LOC_Os11g05562.1	98.8	98.8	100%	99.9	99.9	100%
31. S5a LOC_Os11g29190.1	101.49	101.49	100%			98.6%
32. S18 LOC_Os03g58050.1	100.44	100.44	100%	99.8	99.8	99.2%
33. S18a LOC_Os07g07719.1	100.79	100.79	100%	99.9	?	?
34. S18b LOC_Os07g07770.1	89.03	89.3	99%	99.90	86.72	?

Supplemenatary Table 2- Gene structure of the 34 RPS genes

GENE ID	Chromosome number	Location (bp)	Coded protein	Gene size	GC %	Intron	Exon	Alternative splice forms	Orientation	Gene copies
LOC_Os02g18550	Os2	10822643-10825727	RPS3a	3085	42	5	6	1	3’-5’	3
**LOC_Os01g25610**	Os1	14513321-14515446	RPS4	2126	45	3	4	1	5’-3’	2
**LOC_Os05g30530**	Os4	17693098-17695785	RPS4a	2688	42	3	4	1	5’-3’	1
**LOC_Os01g01060**	Os1	27136-28651	RPS5	1516	46	3	4	1	5’-3’	1
**LOC_Os11g29190**	Os11	16917660-16919756	RPS5a	2097	50	2	3	5	3’-5’	1
**LOC_Os01g12090**	Os1	6583822-6586889	RPS6	3068	40	3	4	1	3’-5’	1
**LOC_Os03g27260**	Os3	15622131-15624526	RPS6a	2396	43	4	5	1	3’-5’	2
**LOC_Os02g21900**	Os2	13025543-13026583	RPS7	1041	39	2	3	1	3’-5’	1
**LOC_Os03g18570**	Os3	10393654-10395829	RPS7a	2176	45	4	5	2	5’-3’	2
**LOC_Os07g43510**	Os7	26036088-26039317	RPS9	3230	42	1	2	1	3’-5’	1
**LOC_Os03g05980**	Os3	2986017-2989284	RPS9-2	3268	40	2	3	2	5’-3’	2
**LOC_Os01g73160**	Os1	42425424-42427258	RPS10	1835	43	3	4	1	3-5’	2
**LOC_Os02g34460**	Os2	20650248-20652056	RPS10a	1809	40	2	3	1	5’-3’	1
**LOC_Os07g38540**	Os7	23153184-23154093	RPS13	910	55	0	1	1	5’-3’	1
**LOC_Os08g02400**	Os8	956309-958681	RPS13a	2373	42	4	5	1	3’-5’	2
**LOC_Os07g08660**	Os7	4460536-4462630	RPS15	2095	43	2	3	1	3’-5’	1
**LOC_Os02g27760**	Os2	16440267-16442516	RPS15a	2250	41	2	3	1	5’-3’	2
**LOC_Os03g01900**	Os3	559227-559947	RPS17	721	61	0	1	1	3’-5’	2
**LOC_Os03g58050**	Os3	33045552-33047410	RPS18	1889	47	2	3	1	3’-5’	2
**LOC_Os07g07719**	Os7	3883541-3885517	RPS18a	1977	41	2	3	1	5’-3’	1
**LOC_Os07g07770**	Os7	3896681-3899826	RPS18b	3146	41	2	3	1	5’-3’	1
**LOC_Os03g31090**	Os3	17714961-17716432	RPS19	1472	45	3	4	1	5’-3’	1
**LOC_Os03g46490**	Os3	26308552-26312894	RPS21	4343	41	2	3	1	3’-5’	1
**LOC_Os03g60400**	Os3	34347552-34349249	RPS23	1698	42	2	3	1	3’-5’	2
**LOC_Os10g20910**	Os10	10591143-10591410	RPS23a	268	44	1	2	1	3’-5’	1
**LOC_Os01g52490**	Os1	30157882-30159775	RPS24	1894	45	4	5	1	3’-5’	3
**LOC_Os08g44480**	Os8	27973018-27974740	RPS25	1723	45	2	3	2	5’-3’	2
**LOC_Os11g05562**	Os11	2522183-2528177	RPS25a	5995	41	2	3	1	5’-3’	1
**LOC_Os01g60790**	Os1	35153694-35155904	S26	2211	44	3	4	1	5’-3’	2
**LOC_Os04g27860**	Os4	16458037-16459244	S27	1208	42	2	3	1	5’-3’	1
**LOC_Os01g22490**	Os1	12645515-12646409	S27a	895	55	0	1	1	3’-5’	2
**LOC_Os10g27174**	Os10	14323500-14324869	S28	1370	47	0	1	1	3’-5’	1
**LOC_Os03g56241**	Os3	32041537-32043408	S29	1872	40	1	2	1	3’-5’	3
**LOC_Os02g56014**	Os2	34295821-34296468	S30	648	52	0	1	1	3’-5’	2

Supplementary table-3- Protein structure of the 34 RPS proteins- the structural features of the 34 RPS proteins including, length, MW, pI, LCR, disordered protein, percentage of alpha and beta strands, ligands, ligand binding residues, GRAVY and other specific interactions are mentioned in the chart.

Protein type	Length (aa)	MW (Da)	p*I*	LCR	Disordered protein (%)	Alpha helix (%)	Beta-Strand (%)	Ligands	Ligand Binding residues	GRAVY	Specific interactions
RPS3a	261	29759.56	9.85	Two(4-19),(247-261)	29	30	28			-0.516	
RPS3-1	233	25999.36	9.63		26	39	24	HEM PROTOPORPHYRIN IX CONTAINING FE	PHE19 GLU22 LEU23 ASN24 MET26 LEU27 TYR36 VAL39 VAL41 ARG42 VAL43 THR44 MET46 ARG47 THR48 GLU49 ILE50 ILE51 ILE52 THR72 VAL74 VAL75 ARG78 PHE79 PHE81 ASN84 GLY85 VAL86 GLU87 LEU88 TYR89 ALA90	-0.195	IF-1
RPS4	265	29807.84	10.13	Two (177-194)(257-265)	14	16	45	NAP (nucleosome assembly protein)	GLY185 GLY186 ARG187 ASN188 ALA217 PHE218 ALA219 THR220 ASN224	-0.417	RNA BINDING SITE
RPS5	200	22226.65	9.74		28	54	9	Mg+	HIS75 TYR148	-0.227	S11 interface, S25 interface,S9 interface, rRNA binding site
RPS6	206	24013.16	4.86	184-206	43	58	8	FMN	LEU8 VAL10 LEU22 ARG25 VAL26 ALA27 ARG29 MET127 MET129 LEU139 LEU142 ASN143 LEU148 TRP151	-0.460	
RPS6a	245	28058.70	10.65	two(85-101)(190-207)	23	44	16	Zn+2	ASP154 ARG156 LEU183	-0.897	IF-1
RPS7	100	11409.51	9.75		31	41	31			0.144	
RPS7a	192	22182.78	9.79		23	41	27	Zn+2	HIS64 THR98	-0.515	
S9	86	9541.93	10.26	One(64-78)	51	20	41			-0.563	
S9-2	195	22673.18	10.29	One (184-195)	38	60	5	Mg+2	ARG55	-0.710	IF-1
S10	179	19910.46	9.81	Two(97-143, (154-177)	51	31	9			-0.892	
S13	151	16934.02	10.54		21	64	0	BCL (non metallich etero	ASP87 PHE90	-0.236	IF-1, RNA binding domain

								gen)			
S15	154	17415.44	10.19		35	31	25	Mg+2	TYR24	-0.470	IF-1
S15a	130	14818.38	9.94		15	28	39	Zn+2	LYS32 ILE35	-0.135	
S17	144	16615.17	10.34		46	65	2	Ca+2	GLU36	-0.730	IF-1, DNA Binding site
S19	146	16386.81	10.04		16	52	4	Mg+2	GLN111	-0.557	
S21	82	9283.40	6.71		34	23	33			-0.662	
S23	142	15660.37	10.43		35	20	26	Mg+2	ASN62	-0.561	aminoacyl-tRNA interaction site (A-site), 18S rRNA interaction site,28S rRNA interaction site,streptomycin interaction site,eEF2 interaction site
S23a	66	7302.83	10.00	One (44-57)	24	30	21			0.309	IF-1
S24	138	15718.58	10.57	Two(127-138), (42-52)	31	36	28	FMN, FAD, Zn+2	ARG15 GLU31 LYS74 THR76	-0.771	
S25	108	12068.04	10.57	One (4-36)	35	56	15			-0.898	
S26	133	14991.30	11.02	Two (4-17), (85-132)						-0.907	
S27	86	9558.14	9.06	One (15-24)	33	14	49			-0.256	
S27a	155	17668.52	9.79	One(74-104)	35	12	31			-0.772	Ubq - E2 interaction site, Ubq - CUE interaction site,Ubq - UCH interaction site
S28	65	7463.76	11.16	One(52-65)	31	17	42			-0.429	RNA binding site
S29	112	12810.93	10.08		61	64	4			-0.623	IF-1
S30	62	6931.16	12.03	One (20-29)	76	61	0			-1.281	
S10a	138	14952.46	9.84	Two(51-98)(109-136)	68	10	12			-1.036	
S4a	263	29662.71	10.15	One(250-263)	11	16	44	NAP	185 GLY 186 GLY 187 ARG 188 ASN 217 GLN 218PHE 219 ALA 220 THR 224 ASN	-0.396	
S13a	151	17115.28	10.53		26	62	0	BCL	ASP87 PHE90	-0.437	
S25a	108	11918.86	10.41		35	56	15			-0.854	
S5a	200	22493.03	9.69		28	55	10	MG	74 HIS 147 TYR	-0.133	
S18	152	17662.58	10.74		15	51	8	MG	32 SER	-0.751	
S18a	126	14740.19	11.00		15	52	2	MG	6 SER	-0.880	
S18b	78	9294.84	11.23		18	37	17			-1.183	

Supplementary table-4 - RPS genes expressing in each tissue specifically.RPS genes with fold levels ≥2 on the log_2 scale were considered as up-regulated.

Embryo	Endosperm	Plumules	Radicles	7 d s	7 d r	Roots	Shoots	Leaves	Panicles	Grains	Flowers	Root-shoot transition
	S3a		S3a	S3a		S3a	S3a	S3a				
S4	S4	S4		S4		S4	S4	S4	S4	S4		S4
								S4a				
S5a	S5a	S5a	S5a	S5a		S5a	S5a	S5a	S5a	S5a		S5a
S6	S6	S6	S6	S6	S6	S6	S6	S6	S6			
		S6a		S6a								
S7a	S7a	S7a		S7a	S7a	S7a	S7a	S7a	S7a	S7a		S7a
S9	S9	S9		S9	S9	S9	S9	S9	S9	S9		S9
S9-2	S9-2	S9-2		S9-2	S9-2	S9-2	S9-2	S9-2	S9-2	S9-2		S9-2
		S10		S10		S10	S10	S10	S10	S10		S10
								S10a				S10a
S13	S13	S13		S13	S13		S13	S13	S13	S13		S13
S13a		S13a	S13a	S13a			S13a	S13a		S13a		S13a
S15		S15		S15		S15	S15	S15	S15	S15		S15
S15a	S15a	S15a		S15a				S15a	S15a	S15a		S15a
S17	S17	S17		S17		S17	S17	S17	S17	S17		S17
S18		S18		S18			S18	S18	S18			S18
S18a		S18a		S18a		S18a	S18a	S18a		S18a		S18a
S18b	S18b	S18b		S18b		S18b	S18b	S18b	S18b	S18b		S18b
				S21				S21				S21
												S23
S24	S24	S24		S24		S24	S24	S24	S24	S24		S24
	S25			S25			S25	S25		S25		S25
S25a		S25a		S25a		S25a	S25a	S25a	S25a			S25a
		S26		S26			S26	S26	S26			S26
S27	S27	S27		S27			S27	S27	S27	S27		S27
				S28	S28							
S29	S29	S29		S29	S29	S29	S29	S29		S29		S29

Supplementary table-5- list of primers with their sequences

Gene name	Forward primer	Reverse primer
S3a	TGAAGCCCATGTGGATGTTA	TCACATGTTGATGCCTGGTT
S4	CAGGTTGAAGTATGCGCTGA	AGCGACCCTTGGTGTCATAG
S4a	AGCGGCATGTTATGGTTGAT	GGACCTTGCAGAGCTTGAAC
S5	ATATCCCTCGCCGACTACCT	TGATCTTCTTGCCGTTGTTG
S5a	CAACGGGAAGAAGATCATGG	GACCCTCCTCAAGGGAGAGA
S6	GCTACGGCATCAAGAAGCTC	CAAGTGCCGTGTCACAAAGT
S6a	CATGAAGCAGGGTGTGCTTA	TCAGTCAAGCCAGGAAGGTC
S7	TGTGCAAGGCATTCAAGAAA	TCCAAGATTCCATCATGAACAG
S7a	AGAAGAAGTTCAGCGGCAAG	GCACCATCCAGACGGTATCT
S9	TTATCAGGCAGCACCACATC	GGCCTTCTGGTTCTTCCTCT
S9-2	GAGCTGTGGCGTGTTCAGTA	AACAGTGAGGGCAAGGACAT
S10	CCCTCAAGAAGTCTGCCAAG	ACCAAAATCACCTGGAGCAC
S10a	GCATTGAGCACCTGAGGAAT	AACCTTGGTCTGTCCCCTTC
S13	AGGTGGAGGAGATGATCGTG	GACGGCCTTCTTGATGAGG
S13a	GTGGTGCTCCGTGACCAG	TGGAGTCCTTGTCCTTCCTG
S15	ATGACCTCGTCCAGCTCTTC	CCGATCATCTCAGGGACAAT
S15a	CCGACCCTCATCAAAGGTTA	TCCTTGACACCAACATCGAA
S17	AACAAGAAGGTGCTGGAGGA	TCCTGGAGCTTGAGGGAGAT
S18	CGACATCGACATGAACAAGC	TCAGCCTCTCAAGGTCATCC
S18a	CTCACCTCCATCAAGGGTGT	ACCTCCCGTCCTTGTAGTCC
S18b	AAGGATGGGAGGTTCTCTCAG	TCTTGGAGACACCGACAGTCT
S19	CAAGATGGAGCTCCCTGAGT	GGACGTGAGCCATTCCTCT
S21	TCAGATGGTGGACCTCTACG	AGAGCACTGTCGGCGTCT
S23	GAGCCATCTTGGCAATGAAT	CGATGAAGTTCAAGCAACCA
S23a	TTTTGGGAGAAAAAGGCTTG	CCTCTGCTTCTTCTTCTCCTTG
S24	GTCTCCAAGGCTGAGCTGAA	CTTGGGCTCGTACTTCTTCG
S25	GGGAAAGCAAAAGGAGAAGG	GATTAGGCCCCGTGTCATTA
S25a	CGGAGGCAAGCAGAAGAAG	CAAGATCCTTGATGGCCTGT
S26	CAAGGCGATCAAGAGGTTTC	GACGATGTGAGCATGGATTG
S27	CGGAGCTGGAGAAGCTCA	CTGGCCTTCCCACCAGTAG
S27a	CGAAGATCCAGGACAAGGAG	CTTGGGCTTGGTGTACGTCT
S28	GGATACCCAGGTCAAACTTGC	CTGGCCTCCCTCTCAGACT
S29	CACTCCAACGTGTGGAACTC	CGGTACTTGATGAAGCCAATG
S30	GAAGGTGAGGGGGCAGAC	GACGAAACGGCGGTTGTACT

SUPPLEMENTARY TABLE 6- List of up-regulated RPL and RPS genes in response to four abiotic stress conditions.

The RPL and RPS genes that are upregulated (≥3 fold) under a particular stress have been listed here. The gene names in blue and red color belong to RPL and RPS gene families respectively and the maximum fold changes of the genes under that particular stress are mentioned in brackets.

**ABA shoot**		**ABA root**	
RPL	RPS	RPL	RPS
L4(24.34), L5(3.8), L6(124.30), L7(13.78), L8(239.07), L10(143.47), L11(8.87), L12(280.52), L13a(16.18), L13b(14.76), L15(16.25), L18a(18.92), L18P(13.13), L19.3(33.48), L22(4.35), L23(23.68), L24(12.5), L24b(17.58), L27(36.0), L28(111.9), L30(63.95), L31(48.24), L32(68.54), L34(21.16), L35(248.90), L38(13.93), L44(25.83), L51(4.31)	S3a(267,2), S4(10.22), S4a(39.54), S5(2513), S5a(55.41), S6(140.87), S6a(187.72), S7(245.67), S7a(12.56), S9(58.97), S9-2 (73.3), S10(71.79), S10a(56.49), S13(76.67), S13a(90.80), S15(28.99), S15a(224.54), S17(186.78), S18(75.63), S18a(278.54), S18b(216.73), S19(153.23), S21(105.17), S23(35.78), S23a(114.56), S24(382.71), S25(106.15), S25a(56.23), S26(32.28), S27(49.96), S27a(302.66), S28(167.88), S29(128.60), S30(113.63)	L5(4.76), L6(3.08), L7(62.58), L8(13.78), L10(6.43), L11(9.52), L12(3.18), L13b(58.14), L14(99.05), L18a(8.06), L18P(32.94), L19.3(52.83), L21.2(41.50), L23(906.26), L24(6.37), L24b(170.11), L26(14.54), L28(164.60), L29(25), L31(10.01), L32(39.87), L34(5.27), L35(16.43), L36(24.77), L37(58.50), L38(75.46), L51(16.22).	S3a(55.89), S4(19.29), S4a(7.08), S5(45.03), S5a(16.32), S6(29.55), S6a(3.31), S7(7.8), S7a(34.01), S9(21.89), S9-2 (4.59), S10(14.77), S10a(9.45), S13(6.66), S15(4.5), S15a(10.14), S17(17.22), S18(7.28), S18b(6.34), S21(21.06), S23(7.72), S23a(5.55), S24(8.76), S25(19.99), S25a(58.42), S26(3.12), S27(26.53), S27a(41.93), S28(9.73), S29(213.33), S30(57.34)

**PEG shoot**		**PEG root**	
RPL	RPS	RPL	RPS
L4(11.73), L5(3.08), L6(145.15), L7(13.91), L8(876.21), L10(19.04), L11(26.71), L12(163.70), L13a(9.32), L13b(15.48), L15(14.45), L18a(9.51), L18P(4.91), L19.3(213.12), L21.2(4.56), L23(34.75), L24(7.87), L24b(3.9), L26(10.68), L27(24.01), L28(39.22), L30(22.09), L31(20.60), L32(34.26), L34(5.44), L35(496.90), L37(30.05), L38(21.45), L44(36.39).	S3a(226.24), S4(467), S4a(641.19), S5(36.84), S5a(8.42), S6(182.15), S6a(61.71), S7(19.53), S7a(3.14), S9(32.99), S9-2(364.83), S10(14.67), S10a(72.60), S13(8.46), S13a(91.59), S15(142.01), S15a(333.14), S17(413.73), S18(134.83), S18a(34.17), S18b(91.7), S19(202.2), S21(58.40), S23(6.05), S23a(218.64), S24(262.88), S25(110.35), S25a(41.51), S26(70.88), S27(131.12), S27a(251.68), S28(264.44), S29(33.12), S30(127.31)	L4(6.11),L7(8.42), L8(36.97), L10(4.71), L11(8.09), L12(36.03), L13b(22.88), L14(19.53), L18a(5.28), L18P(29.56), L19.3(19.68), L21.2(27.58), L23(48.31), L24(4.02), L24b(13.38), L26(18.25), L27(13.05), L28(19.96), L29(65.33), L31(7.57), L32(15.23), L34(30.72), L35(12.10), L36(30.72), L37(57.82), L38(57.09), L51(22.46)	S3a(13.56), S4a(15.29), S5(47.36), S5a(5.55), S6(24.11), S6a(12.40), S7(18.66), S7a(36.64), S9-2 (14.98), S10(3.25), S10a(11.54), S13(10.53), S15(4.58), S15a(8.97), S17(20.17), S18(3.07), S18b(4.9), S21(29.51), S23(33.39), S23a(7.91), S24(5.42), S25(9.43), S25a(230.74), S26(3.40), S27(22.27), S28(32.11), S29(146.67), S30(9.07)

**NaCl shoot**		**NaCl root**	
RPL	RPS	RPL	RPS
L4(40.24), L5(5.29), L6(27.13), L7(10.62), L8(48.49), L10(60.93), L11(18.13), L12(170.39), L13a(20.55), L13b(10.08), L18a(3.22), L18P(4.89), L19.3(48.40), L23(17.61), L27(10.50), L32(4.94), L35(17.04), L37(3.47)	S3a(394.95), S4(107.34), S4a(754.56), S5(64.99), S5a(233.41), S6(132.97), S6a(47.95), S7(135.30), S7a(5.44), S9(325.91), S9-2(1357.75), S10(380.49), S10a(129.63), S13(364.99), S13a(187.85), S15(88.46), S15a(724.23), S17(252.99), S18(885.39), S18a(1068.83), S18b(318.11), S19(332.04), S21(96.45), S23(3.37), S23a(439.57), S24(238.23), S25(129.19), S25a(91.95), S26(143.50), S27(552.5), S27a(1420.60), S28(543.29), S29(47.44), S30(65.93)	L6(5.09), L7(139.01), L8(13.56), L10(4.4), L11(10.20), L12(7.79), L13b(4.62), L14(51.41), L18P(32.98), L19.3(25.54), L21.2(106.62), L23(41.92), L24(4.19), L24b(11.00), L26(3.12), L28(18.98), L29(30.50), L31(13.14), L32(12.93), L34(3.35), L35(22.74), L36(25.45), L37(26.13), L38(69.29), L51(16.45)	S3a(17.0), S4(5.32), S4a(14.40), S5(8.65), S5a(36.43), S6(5.44), S7(32.01), S7a(), S9(3.75), S9-2(11.27), S10(8.40), S10a(19.8), S13(7.33), S13a(53.19), S15(3.18), S18a(3.91), S18b(84.05), S21(13.65), S23(164.02), S23a(5.46), S24(32.20), S25(14.58), S25a(34.99), S26(6.94), S27(22.45), S27a(9.35), S28(33.77), S29(50.48), S30(15.40)
**H_2O_2 shoot**		**H_2O_2root**	
RPL	RPS	RPL	RPS
L4(3.9), L5(15.25), L6(7.24), L7(4.07), L14(12.20), L18a(62.41), L19.3(7.7), L21.2(3.25), L22(21.25),	S3a(83.86), S4(29.68), S4a(487.00), S5a(33.87), S6(53.10), S6a(69.88), S7(180.48), S7a(15.94),	L4(126.92), L5(18.09), L6(4.92), L7(8.16), L8(1.25), L13a(222.42), L14(57.69), L15(215.27), L18a(43.28),	S3a(3.46), S4a(10.73), S5(22.29), S5a(14.24), S6(14.35), S7(3.00), S7a(9.80), S9-2(5.18), S10(5.65),

L24(36.86), L28(81.50), L29(3.10), L30(3.80), L31(18.96), L32(11.61), L34(8.6), L37(14.58), L38(15.93), L44(14.47), L51(8.48)	S9(269.65), S9-2(329.89), S10(93.11), S10a(87.74), S13(69.70), S13a(137.24), S15(51.09), S15a(298.48), S17(154.85), S18(187.73), S18a(491.19), S18b(62.92), S19(71.76), S21(81.90), S23(3.08), S23a(164.71), S24(66.26), S25(58.89), S25a(155.55), S26(47.17), S27(66.18), S27a(146.72), S28(137.66), S29(37.30), S30(30.58)	L18P(6.36), L19.3(4.36), L21.2(17.24), L22(80.45), L23(18.12), L24b(194.48), L26(148.92), L27(5.95), L29(6.27), L30(188.155), L31(13.89), L32(25.11), L34(241.73), L35(142.88), L36(33.39), L37(47.87), L38(26.64), L44(6.86), L51(343.61)	S10a(11.69), S15(6.49), S15a(4.29), S17(7.78), S18(6.04), S18b(4.56), S21(11.50), S23(14.99), S24(23.09), S25(7.79), S25a(34.66), S27(23.34), S28(78.78), S29(16.65), S30(9.01)

Supplementary Table 7------Tissue specific elements observed in the upstream sequences of 34 rice RPS genes as found from PlantCARE database

GENE	TISSUE SPECIFIC ELEMENT	REPORTED FUNCTION	POSITION IN THE UPSTREAM SEQUENCE	SEQUENCE OF THE ELEMENT
S3a	RY-element	CARE element involved in seed specific regulation	557	CATGCATG
S4	Skn-1 motif	CARE required for endosperm expression	122,269	GTCAT
s5	CAT-box	CARE required for meristem expression	7	GCCACT
	Skn-1 motif	CARE required for endosperm expression	957	GTCAT
S6	CAT-box	CARE required for meristem expression	994	GCCACT
	Skn-1 motif	CARE required for endosperm expression	522	GTCAT
S6a	AC-II	Negative regulation in phloem expression & restricts vascular expression to xylem	76	CTCACCAACCCC
S7	CAT-box	CARE required for meristem expression	169	GCCACT
	CCGTCC- box	CARE related to meristem specific activation	809	CCGTCC
	GCN4 motif	CARE required for endosperm expression	611,727	TGAGTCA
	Skn-1 motif	CARE required for endosperm expression	10,031,009	GTCAT
S7a	ATGCAAAT motif	Associated to the TGAGTCA motif(which is GCN4 motif responsible for endosperm expression)	703,854	ATACAAAT
	Skn-1 motif	CARE required for endosperm expression	349,469	GTCAT
S9	CAT-box	CARE required for meristem expression	482	GCCACT
	Skn-1 motif	CARE required for endosperm expression	749	GTCAT
S9-2	CAT-box	CARE required for meristem expression	575	GCCACT
	CCGTCC- box	CARE related to meristem specific activation	940	CCGTCC
	GCN4 motif	CARE required for endosperm expression	720	TGAGTCA
	dOCT	CARE related to meristem specific activation	750,771	CaCGGATC
s10	Skn-1 motif	endosperm expression	344,	GTCAT
S13	CCGTCC- box	Meristem specific activation	583,	CCGTCC
	Skn-1 motif	endosperm expression	381, 516, 427	GTCAT
	as-2-box	shoot specific expression and light responsiveness	848	GATAATGATG
S15	CAT-box	meristem expression	76, 870	GCCACT
	CCGTCC- box	meristem activation	105	CCGTCC

	Skn-1 motif	endosperm expression	163, 566, 342, 768,188,	
			729, 505,	GTCAT
S15a	CCGTCC- box	meristem activation	88, 847	CCGTCC
	Skn-1 motif	endosperm expression	475	GTCAT
S17	No site			
S19	CCGTCC- box	meristem activation	211, 980, 798.	CCGTCC
	Skn-1 motif	endosperm expression	306, 719, 529, 901	GTCAT
S21	CCGTCC- box	meristem activation	828, 832	CCGTCC
	GCN4 motif	endosperm expression	553, 926	TGAGTCA
	ATGCAAAT motif	Associated to the TGAGTCA motif(which is GCN4 motif responsible for endosperm expression)	432	ATACAAAT
S23	CAT-box	meristem expression	448	GCCACT
	Skn-1 motif	endosperm expression	498, 749, 561,802	GTCAT
	dOCT	Meristem specific activation	742	CaCGGATC
S23a	Skn-1 motif	endosperm expression	284	GTCAT
S24	Skn-1 motif	endosperm expression	337, 872	GTCAT
S25	CAT-box	related to meristem expression	701	GCCACT
	CCGTCC- box	meristem expression	67, 962. 947	CCGTCC
	GCN4 motif	endosperm expression	446	TGAGTCA
S26	CAT-box	meristem expression	367, 677, 542	GCCACT
S27	CCGTCC- box	Meristem specific activation	890	CCGTCC
S27a	CCGTCC- box	Meristem specific activation	65, 216	CCGTCC
	Skn-1 motif	endosperm expression	751, 841	GTCAT
	AC-II	enhanced xylem expression and repressed phloem expression	253	CCACCAACCCCC
S28	Skn-1 motif	endosperm expression	591	GTCAT
S29	Skn-1 motif	endosperm expression	651	GTCAT
S30	CAT-box	meristem expression	603	GCCACT
S5a	Skn-1 motif	endosperm expression	765	GTCAT
	as-2-box	shoot specific expression and light responsiveness	780	GATAATGATG
S18	GCN4 motif	endosperm expression	532	TGAGTCA
S18a	AC-II	enhanced xylem expression and repressed phloem expression	562	CCACCAACCCCC
	GCN4 motif	endosperm expression	850	TGAGTCA
	Skn-1 motif	endosperm expression	854, 853, 763	GTCAT
s18b	Skn-1 motif	CARE required for endosperm expression	131,277,201,695	GTCAT
	as-2-box	shoot-specific expression and light responsiveness	447,503,477	GATAatGATG
S10a	CAT-box	meristem expression	31	GCCACT
	Skn-1 motif	endosperm expression	410	GTCAT
S4a	CAT-box	meristem expression	44,118	GCCACT
	Skn-1 motif	endosperm expression	5,37,589	GTCAT
S13a	Skn-1 motif	endosperm expression	3	GTCAT
S25a	CAT-box	meristem expression	125	GCCACT
	as-2-box	shoot specific expression and light responsiveness	324	GATAATGATG

Supplementary table-8- Domain analysis of the ribosomal proteins small subunits-

GENE	DOMAIN	POSITION	SEQUENCE	LCR	POSITION	SEQUENCE
S3a	Ribosomal_S3Ae	15-221	GSKKKTVDPFSKKDWYDIKAP TVFSVRNIGKTLVSRTQGTKI ASEGLKHRVFEVSLADLQ NDEDQAYRKVRLRAEDVQGR NVLTNFWGMSFTTDKLRSLV KKWQTLIEAHVDVKTTDNYM LRLFCIGFTKRRPNQVKRTC YAQASQIRQIRRKMVEIMAN QASTCDLKELVSKFIPEVIG KEIEKSTSSIFPLQNVFIRK VKILKAP	1	247-261	AETDEAVAGEVAAAE
				2	4-19	GKNKRISKGRKGSKKK
S4	RS4NT	3-39	RGLKKHLKRLNAPKHWMLDKL GGAFAPKPSSGPHKSR	1	177-194	VGNVVMVTGGRNTGRVGV
	S4	42-106	LPLILIIRNRLKYALTYREVISIL MQRHVLVDGKVRTDKTYPAGF MDVISIPKTGENYRL LYDTK	2	257-265	AAAQAAANA
	40S_S4_C	212-259	DALGHAFATRLGNVFTIGKGNK PWVSLPKGKGIKLSIIEEQRKRD AAA			
	Ribosomal_S4e	95-169	TGENYRLLYDTKGRFRLQSVKD EDAKFKLCKVRSVQFGQKGIPY LNTYDGRTIRYPDPLI KANDTIKIDLETNKI			
	KOW	177-211	VGNVVMVTGGRNTGRVGVIKN REKHKGSFETIHVE			
S5	Ribosomal_S7	47-200	HTAGRYSAKRFRKAQCPLVERL TNSLMMHGRNNGKKIMAVRIV KHAMEIIHLLTDANPIQ VIVDAIINSGPREDATRIGSAGA VRRQAVDISPLRRVNQAIYLLTT GARESAFRNIKTIA ECLADELINAAKGSSNSYAIKKK DEIERVAKANR	----	-----	----
S6	Ribosomal_S6	3-73	LYDCMLLVKPLVTREAMAELV GRVARRAYQRNGVVTDVKSFG TICLGYGIKKLDGRHFKE YQDYSGWVAQK	1	184-206	DEASDVDEYDDDDDDYEYEIDEE
	Ribosomal_S6	104-155	GTLKTHFVTRHLNVSAIEGQLM QMTMMVPPSFTQELHYLNKED RLLRWLVVK			
	SPT2	132-206	PPSFTQELHYLNKEDRLLRWLV VKHRDAVYGVEFINEDDGRRE MTDFRYRTKDEASDVDE YDDDDDDYEYEIDEE			
S6a	Ribosomal_S6e	1-128	MKFNIANPTTGCQKKLEIDDDQ KLRAFYDKRISQEVSGDALGEE FKGYVFKIMGGCDKQG FPMKQGVLTSGRVRLLLHRGTP CFRGYGRRDGERRRKSVRGCIV SQDLSVINLVIVKKGD NDLPGLTD	1	85-101	RGYGRRDGERRRKSVRG
	Coiled coil	187-220	LTLQRKRARIAQKKQRIAKKKS EAAEYQKLLAQR	2	190-207	QRKRARIAQKKQRIAKKK
S7	Ribosomal_S7e	9-97	ASRLIDLVVVIHVLYHLCKAFK KIHVRLVKELEKKFSGKDVVFD ATRRIVRPLNKGSAVH HPRTRTLITVHDGILEDVVSQLR LLGSIS	----	----	----
GENE	DOMAIN	POSITION	SEQUENCE	LCR	POSITION	SEQUENCE
	TGc	7-55	FSASRLIDLVVVIHVLYHLCKAF KKIHVRLVKELEKKFSGKDVVF DATR			
S7a	Ribosomal_S7e	7-188	KIQKEKGLEPSEFEDSVAQAFFD LENGNQELKSELKDLYINNAVQ MDIAGNRKAVVIHVP YRLRKAFKKIHVRLVRELEKKF SGKDVVIVATRRIVRPPKKGSA VQRPRTRTLTAVHDGI LEDVVYPAEIVGKRIRYRLDGA KVIKIFLDPKERNNTEYKLETFS AVYRRLCGKDVAFEY PM	----	----	----
S9	S4	1-61	MAKSIHHARVLIRQHHIRVGRQ LVNIPSFIVRLESEKHIAFSLTSPL GGSPAVRVKRKNQ K	1	64-78	GGGGDGEEEEEKELG

S9-2	Ribosomal_S4	8-108	RNYGKTFKKPRRPYEKERLDAE LKLVGEYGLRCKRELWRVQYA LSRIRNNARHLLTLDEK NPRRIFEGEALLRRMNRYGLLA DGQNKLDYVLALTVENFLA	1	184-195	GGGGDGEEEDEE
	S4	109-179	RRLQTLVFKAGMAKSIHHARVL IRQRHIRVGRQIVNIPSFMVRVE SEKHIDFSLTSPFGG GPPGRVKRKNQ			
S10	S10_plectin	3-95	ISKKNRREICKYLFHEGVLYAK KDYNLAKHPKVDVPNLEVIKL MQSFKSKEYVRETFSWQ HYYWYLTNDGIEHLRSYLNLPS EVVPNTLKKSA	1	97-143	PPSRPFGSGPPGDRPRGPPRFEGDRPRF GDRDGYRGGPRGAPGDFGG
				2	154-177	PSFRGSRPGFGRGGGGAFGGGASS
S13	Ribosomal_S13_N	1-60	MGRMHSSGKGMSCSVLPYRRA APAWVKTSASEVEEMIVRVAK KGQLPSQIGAILRDAHAV	----	----	----
	Ribosomal_S15	70-149	KILRVLKSRGLAPEVPEDLYFLI KKAVAMRKHLERNRKDKDTKF RLILVESRVHRLTRYY RLAKKIPAFFKYDSTTASTL			
S15	Ribosomal_S19	56-137	RGLKRKPMALIKKLRKAKKDAP AGEKPEPVRTHLRNMIIVPEMIG SIVGVYNGKTFNQVE IKPEMIGHYLAEFSISYKPVKH	----	----	----
S15a	Ribosomal_S8	5-130	SVLNDALKTMYNAEKRGKRQV MIRPSSKVIIKFLIVMQKHGYIGE FEFVDDHRSGKIVVE LNGRLNKCGVISPRFDVGVKEIE SWTARLLPSRQFGYIVLTTSAGI MDHEEARRKNVGGK VLGFFY	----	----	----
S17	Ribosomal_S17e	1-118	MGRVRTKTVKKTSRQVIEKYYS RMTLDFHTNKKVLEEVSILPSKR LRNKVAGFSTHLMRR IQRGPVRGISLKLQEEERERRMD FVPDRSALEVDDIRVDKETLDM LTSLGMADLPGVV	----	----	----
S19	Ribosomal_S19e	8-143	TVKDVNPHEFVKAYSAHLKRSG KMELPEWVDIVKTARFKELPPY DPDWYYTRAASIARKI YLRQGIGVGGFQKIYGGRQRNG SRPPHFCKSSGAISRNILQQLQK MGIIDVDPKGGRLIT SQGRRDLDQVAGRVDV	----	----	----
	Coiled coil	106-133	HARKAEMRTRMKKVLKALEKL RKKADAT			
S21	Ribosomal_S21e	1-78	MQNEEGQMVDLYVPRKCSTTN RIITAKDHASVQINIGHVDENGL YDGRFTTFALSGFIRA QGDADSALDRLWQKRKAE	----	----	----
S23	Ribosom_S12_S23	8-141	GAGRKLKTHRRNQRWADKAY KKSHLGNEWKKPFAGSSHAKGI VLEKIGIEAKQPNSAIRK CARVQLVKNGKKIAAFVPNDG CLNFIEENDEVLIAGFGRKGHAV GDIPGVRFKVVKVSGV SLLALFKEKKEKPR	----	----	----
S23a	Ribosom_S12_S23	8-66	GLYDGIFRKLLLSMDEVLIVGFG WKGHALGDIPGVRLKVVKVSA VSLLALFKEKKKQRS	1	44-57	LKVVKVSAVSLLAL
S24	Ribosomal_S24e	29-108	VLEVIHPGRPNVSKAELKEKLA KLYEVKDANCIFVFKFRTHFGG GKSTGFGLIYDNLDAA KKYEPKYRLIRNGLATKVEK	1	127-138	KKTKAGDAGKKK
				2	42-52	KAELKEKLAKL
S25	Ribosomal_S25	4-105	KKDKAPPPSSKPAKSGGGKQKK KKWSKGKQKEKVNNSVLFDQA TYDKLLSEVPKYKQITP SVLSERLRINGSLARRAINDLMT RGLIRMVSVHSSQQIYTRA	1	4-36	KKDKAPPPSSKPAKSGGGKQKKKKW SKGKQKEK
S26	Ribosomal_S26e	1-108	MTFKRRNGGRNKHGRGHVKYI RCSNCAKCCPKDKAIKRFQVRN IVEQAAIRDVQEACVHD GYVLPKLYAKVHHCVSCAIHAH IVRVRSRENRRDRRPPERFRRRE DRP	1	4-17	KRRNGGRNKHGRGH
				2	85-132	RVRSRENRRDRRPPERFRRREDRPQGP RPGGGAPAPGGAAAPAPNVAR
S27	Ribosomal_S27e	30-84	PNSFFMDVKCQGCFNITTVFSHS QTVVVCPGCQTVLCQPTGGKA RLTEGCSFRRK	1	15-24	ELEKLKHKKK

S27a	UBQ	1-72	MQIFVKTLTGKTITLEVESSDTIDNVKAKIQDKEGIPPDQQRLIFAGKQLEDGRTLADYNIQKESTLHLVLR	1	74-104	RGGAKKRKKKTYTKPKKQKHKHKKVKLAVLQ
	Ribosomal_S27	102-147	VLQFYKVDDATGKVTRLRKECPNAECGAGTFMANHFDRHYCGKCGLT			
GENE	DOMAIN	POSITION	SEQUENCE	LCR	POSITION	SEQUENCE
	Rad60-SLD	1-71	MQIFVKTLTGKTITLEVESSDTIDNVKAKIQDKEGIPPDQQRLIFAGKQLEDGRTLADYNIQKESTLHLVL			
S28	Ribosomal_S28e	3-64	TQVKLAVVVKVMGRTGSRGQVTQVRVKFLDDQNRLIMRNVKGPVREGDILTLLESEREARRL	1	52-65	LTLLESEREARRLR
S29	Ribosomal_S14	6-56	VWNSHPKNYGPGSRVCRVCGNPHGLIRKYGLMCCRQCFRSNAKDIGFIKYR	----	----	----
S30	Ribosomal_S30	3-60	KVHGSLARAGKVRGQTPKVAKQDKKKKPRGRAHKRMQYNRRFVTAVVGFGKKRGPNSS	1	20-29	KVAKQDKKKK
S10a	S10_plectin	1-50	MQSFKSKEYVRETFSWQYYYWYLTNDGIEHLRNYLNLPSEIVPATLKKSA	1	51-98	RPPGRPFGSGPPGDRPRGPPRFEGDRPRFGDRDGYRGGPRGAPGDFGG
	AT_hook	50-62	ARPPGRPFGSGPP	2	109-136	PSFRSSGGRPGFGRGGGGGFGAGPTSSS
S4a	RS4NT	3-39	RGLKKHLKRLNAPSHWMLDKLGGAFAPKPSSGPHKAR	1	181-194	VMVTGGRNTGRVGV
	S4	42-106	LPLILILRNRLKYALTYREVISILMQRHVMVDGKVRTDKTYPAGFMDVVSIAKTGENFRLLYDTK	2	250-263	EEARKRNAEAAAEA
	KOW	174-201	KFDVGNIVMVTGGRNTGRVGVIKSREKH			
	40S_S4_C	212-259	DALGHQFATRMGNVFTIGKERKPWVSLPKGKGIKLSIIEEARKRNAEA			
	Ribosomal_S4e	95-169	TGENFRLLYDTKGRFRLHSIKDEDAKFKLCKVRSVQFGQKGIPFLNTNDGRTIRYPDPLIKANDTIKIDLETNKI			
S13a	Ribosomal_S13_N	1-60	MGRMHSRGKGISSSAIPYKRTPPSWVKTAAADVEEMIMKAAKKGQMPSQIGVVLRDQHGI	----	----	----
	Ribosomal_S15	70-149	KILRILKAHGLAPEIPEDLYFLIKKAVAIRKHLERNRKDKDSKFRLILVESRIHRLARYYKRTKKLPPTWKYESTTASTL			
S25a	Ribosomal_S25	4-105	KKDKAPPPSSKPAKSGGGKQKKKKWSKGKQKEKVNNSVLFDKATYDKLLSEVPKYKQITPSVLSERLRINGSLARQAIKDLESRGAIRVVSVHSSQLIYTRA	1	4-36	KKDKAPPPSSKPAKSGGGKQKKKKWSKGKQKEK
S5a	Ribosomal_S7	46-185	HTAGRYSAKRFRKAQCPIVERLTNSLMMHGRNNGKKIMAVRIVKHAMEIIHLLTDANPIQVIVDAIINSGPREDATRIGSAGAVRRQAVDISPLRRVNQAIYLLTTGARESAFRNIKTIAECLADELINAAKGSSNRYNT	----	-----	----
	HLH	50-104	RYSAKRFRKAQCPIVERLTNSLMMHGRNNGKKIMAVRIVKHAMEIIHLLTDANPI			
	CARD	73-170	MHGRNNGKKIMAVRIVKHAMEIIHLLTDANPIQVIVDAIINSGPREDATRIGSAGAVRRQAVDISPLRRVNQAIYLLTTGARESAFRNIKTIAECLAD			
GENE	DOMAIN	POSITION	SEQUENCE	LCR	POSITION	SEQUENCE
	ANTAR	125-173	SAGAVRRQAVDISPLRRVNQAIYLLTTGARESAFRNIKTIAECLADELI			

S18	Ribosomal_S13	14-142	RLLNTNVDGKQKIMFALTSIKG VGRRFSNIACKKADIDMNKRAG ELTPEELERLMTVVAN PRQFKVPDWFLNRKKDYKDGR FSQVVSNALDMKLRDDLERLK KIRNHRGLRHYWGLRVRG QHTKTTGRR	1	136-148	TKTTGRRGKTVGV
S18a	Ribosomal_S13	1-116	MFALTSIKGIGRRFSNIACKKAD IDMNKRAGELTPEELERLMTVV ANPRQFKVPDWFLNR KKDYKDGRFSQVVSNALDMKL RDDLERLKKIRNHRGLRHYWG LRVRGQHTKTTGRR	1	110-122	TKTTGRRGKTVGV
	HALZ	20-61	KKADIDMNKRAGELTPEELERL MTVVANPRQFKVPDWFLNRK			
	SANT	29-170	RAGELTPEELERLMTVVANPRQ FKVPDWFLNRKKDYKDGRFSQ VVSNALDMKLRDDLERL KKIRNHRGLRHYWGLRVRG			
	LYZ1	31-117	GELTPEELERLMTVVANPRQFK VPDWFLNRKKDYKDGRFSQVV SNALDMKLRDDLERLKK IRNHRGLRHYWGLRVRGQHTK TTGRRG			
	GLUCA	64-89	YKDGRFSQVVSNALDMKLRDD LERLK			
S18b	Ribosomal_S13	3-68	PYMSWILPERKKDYKDGRFSQV VSNALDMKLRDDLERLKKIRNH RGLRHYWGLRVRGQHT KTTGRR	1	62-74	TKTTGRRGKTVGV
	GLUCA	16-81	YKDGRFSQVVSNALDMKLRDD LERLK			

Supplementary table-9 This contains the list of the 34 RPS genes along with their paralogous members in rice.

GENE NAME	GENE SELECTED	OTHER PARAOGOUS MEMBERS IN RICE
1. S3a	LOC_Os02g18550	LOC_Os03g10340<br>LOC_Os12g21798
2. S4	LOC_Os01g25610	LOC_Os02g01560
3. S5	LOC_Os01g01060.1	
4. S6	LOC_Os01g12090.1	
5. S6a	LOC_Os03g27260.1	LOC_Os07g42950
6. S7	LOC_Os02g21900.1	
7. S7a	LOC_Os03g18570.1	LOC_Os03g18580<br>LOC_Os05g27940
8. S9	LOC_Os07g43510.1	
9. S9-2	LOC_Os03g05980.1	LOC_Os11g38959
10. S10	LOC_Os01g73160.1	LOC_Os04g35090
11. S13	LOC_Os07g38540.1	
12. S15	LOC_Os07g08660.1	
13. S15a	LOC_Os02g27760.1	LOC_Os07g10720
14. S17	LOC_Os03g01900.1	LOC_Os10g27190
15. S19	LOC_Os03g31090.1	
16. S21	LOC_Os03g46490.1	
17. S23	LOC_Os03g60400.1	LOC_Os01g61814
18. S23a	LOC_Os10g20910.1	
19. S24	LOC_Os01g52490.1	LOC_Os02g13530<br>LOC_Os06g36160
20. S25	LOC_Os08g44480.1	LOC_Os09g39540
21. S26	LOC_Os01g60790.1	LOC_Os05g39960
22. S27	LOC_Os04g27860.1	
23. S27a	LOC_Os01g22490.1	LOC_Os05g06770
24. S28	LOC_Os10g27174.1	
25. S29	LOC_Os03g56241.1	LOC_Os11g41610<br>LOC_Os12g32380
26. S30	LOC_Os02g56014.1	LOC_Os06g07580
27. S10a	LOC_Os02g34460.2	
28. S4a	LOC_Os05g30530.1	
29. S13a	LOC_Os08g02400.1	LOC_Os08g02410
30. S25a	LOC_Os11g05562.1	

31. S5a	LOC_Os11g29190.1	
32. S18	LOC_Os03g58050.1	LOC_Os07g07709
33. S18a	LOC_Os07g07719.1	
34. S18b	LOC_Os07g07770.1	
